# The Impact of Modern Technologies on Molecular Diagnostic Success Rates, with a Focus on Inherited Retinal Dystrophy and Hearing Loss

**DOI:** 10.3390/ijms22062943

**Published:** 2021-03-14

**Authors:** Suzanne E. de Bruijn, Zeinab Fadaie, Frans P. M. Cremers, Hannie Kremer, Susanne Roosing

**Affiliations:** 1 Department of Human Genetics, Radboud University Medical Center, 6500 HB Nijmegen, The Netherlands; Suzanne.deBruijn@radboudumc.nl (S.E.d.B.); Zeinab.Fadaie@radboudumc.nl (Z.F.); Frans.Cremers@radboudumc.nl (F.P.M.C.); 2 Donders Institute for Brain, Cognition and Behaviour, Radboud University Medical Center, 6500 HB Nijmegen, The Netherlands; Hannie.Kremer@radboudumc.nl; 3 Department of Otorhinolaryngology, Radboud University Medical Center, 6500 HB Nijmegen, The Netherlands

**Keywords:** inherited hearing loss, inherited retinal dystrophies, genetic diagnostics, diagnostic yield, next-generation sequencing, third-generation sequencing, variant interpretation

## Abstract

The identification of pathogenic variants in monogenic diseases has been of interest to researchers and clinicians for several decades. However, for inherited diseases with extremely high genetic heterogeneity, such as hearing loss and retinal dystrophies, establishing a molecular diagnosis requires an enormous effort. In this review, we use these two genetic conditions as examples to describe the initial molecular genetic identification approaches, as performed since the early 90s, and subsequent improvements and refinements introduced over the years. Next, the history of DNA sequencing from conventional Sanger sequencing to high-throughput massive parallel sequencing, a.k.a. next-generation sequencing, is outlined, including their advantages and limitations and their impact on identifying the remaining genetic defects. Moreover, the development of recent technologies, also coined “third-generation” sequencing, is reviewed, which holds the promise to overcome these limitations. Furthermore, we outline the importance and complexity of variant interpretation in clinical diagnostic settings concerning the massive number of different variants identified by these methods. Finally, we briefly mention the development of novel approaches such as optical mapping and multiomics, which can help to further identify genetic defects in the near future.

## 1. Introduction

In previous decades, different methods for disease gene identification have been established and successfully employed. All these technologies have significantly contributed to identifying the large number of genes that are associated with inherited forms of hearing loss (HL) (>150 genes) [1] and retinal dystrophy (RD) (>270 genes) [2]. HL is the most common sensory disorder, and it affects 466 million people worldwide [3]. The impact of HL is generally severe as it has profound consequences, especially on mental health, including anxiety, depression, and social isolation [4,5]. HL can be explained by both congenital or acquired causes and displays high clinical heterogeneity in the age of onset, progression, and severity, amongst others [6]. RD represents a group of clinically heterogeneous disorders that involves the death or dysfunction of photoreceptor cells in the retina. Collectively, RDs affect 2 million people worldwide [7]. Generally, three different types of RDs can be distinguished based on the primarily affected cell type: (1) the rod photoreceptors (e.g., retinitis pigmentosa or choroideremia), (2) the cone photoreceptors (e.g., macular and/or cone dystrophies), and (3) more generalized types of RDs that involve both photoreceptor types (Leber congenital amaurosis, cone-rod dystrophies).

Especially for heterogeneous conditions such as RD and HL, the introduction of next-generation sequencing (NGS) techniques has led to the assumption that, soon, all HL- and RD-associated genes will be identified. Nevertheless, the diagnostic yield suggests a significant portion of missing heritability, which can potentially be explained by unrecognized disease genes or missed variants [8,9]. To provide a genetic diagnosis for all inherited cases, it has become evident that there is no single technique that can serve as the gold standard. To be able to detect and interpret all genetic variations of the human genome, classical methods such as linkage analysis or homozygosity mapping should be combined with novel state-of-the-art techniques [10,11].

The observed high genetic heterogeneity is not unique for these inherited sensory disorders; they have also been described for other inherited disorders, including intellectual disability, ciliopathies, and inherited susceptibility for cancer [12,13,14]. Although, in general, disease gene identification strategies applied in these fields rely on the same principles and have undergone a similar development, an optimal diagnostic strategy depends heavily on key factors such as evolutionary pressure and the involvement of multifactorial versus monogenic causes. For example, for intellectual disability, de novo causes are more frequent due to a strong reduction of reproductive fitness; this impacts the optimal diagnostic strategy. This review focuses on the identification of monogenic causes of inherited HL and RD.

In this review, we aim to provide an overview of the development of techniques that have enabled disease gene discovery throughout the years (Section 2). Additionally, we evaluate and highlight the complexity and different aspects of candidate variants and candidate gene interpretation (Section 3). Finally, we describe recent and upcoming improvements and innovations of existing technologies and the development of novel technologies in the field (Section 4).

## 2. Identification of Genes Associated with Hearing Loss and Retinal Dystrophy 

### 2.1. Linkage Analysis

The first HL- and RD-associated genes were identified using linkage analysis and candidate gene strategies in the early 90s [15,16,17]. Examples of candidate gene approaches include analysis of candidate-disease-associated genes based on their function, gene expression, or animal model studies (discussed in [18]). Linkage analysis was used to pinpoint a genomic region of interest likely to encompass the disease gene. The strategy is based on the key principle that a disease haplotype is shared between affected individuals within a family but is not present in unaffected individuals. The shared haplotype cosegregates with the disease, according to the observed mode of inheritance. Initially, linkage regions were mapped using laborious genotyping of polymorphic microsatellite markers, but the process was optimized when microarray technologies became available. Microarrays, such as SNP arrays, allow rapid genotyping of thousands of single nucleotide polymorphisms (SNPs) that are present across the genome and have a variant allele frequency higher than 1% in the healthy population. The higher the density of the SNPs on the array and the more SNPs that reside within the region showing linkage disequilibrium, the more precise the determination of a possible disease haplotype is. The distance between two SNPs can be expressed in centimorgans (cM). One cM is defined as the distance between chromosomal positions that have a 1% chance of being separated by chromosomal recombination during meiosis. A logarithm of the odds (LOD) score can be calculated to estimate the odds that two loci, or a locus and a disease-associated gene, are located at an assumed distance from each other (expressed as the recombination fraction theta). A LOD score of 3.3 or higher is considered evidence for linkage in a genome-wide manner, with a probability of 95% [19]. Nowadays, several tools (e.g., GENEHUNTER [20] and PLINK [21]) are available to calculate the LOD score and identify a linkage region. However, large family pedigrees and sufficient participating family members are required to reach a statistically significant linkage. When a disease-associated locus is defined, Sanger sequencing can be performed to evaluate the genomic region for causative variants. In this way, the linkage analysis strategy has been applied very effectively for disease gene identification for many years (reviewed in [18,22]). Despite the introduction of higher-throughput sequencing techniques, SNP genotyping can still be very useful to determine regions of genotype-sharing even in small families, especially to reduce the number of candidate variants.

### 2.2. Homozygosity Mapping

Genome-wide homozygosity mapping has proven to be a powerful tool to identify disease-associated genes for autosomal recessive disorders. For both inherited HL and RD, a significant number of disease-associated genes were identified using this strategy [18,22]. In consanguineous families, a pathogenic variant is often present in a homozygous state as it is inherited from a recent common ancestor (grandparent or great-grandparent). Homozygosity mapping, which is often performed using SNP arrays, can be used to determine regions that contain consecutive homozygous variants [21,23]. Although the average size of homozygous stretches is larger in consanguineous families (typically between 30 to 100 megabase (Mb)-sized regions [24,25]), several studies have indicated that this method is also an effective tool for nonconsanguineous families (1–30 Mb-sized homozygous regions [24,25,26]). *EYS* is one of the most frequently mutated genes in RD, and it was identified using homozygosity mapping in a nonconsanguineous family [27,28]. Other examples of disease gene identification using homozygosity mapping in nonconsanguineous families include *PDE6C* [29], which is associated with RD, and *OTOG* [30] and *MYO15A* [31], which are implicated for HL. The size of a homozygous disease-associated haplotype decreases over subsequent generations due to meiotic recombination. Well-characterized families and detailed phenotypic information are prerequisites for the successful application of this technique.

### 2.3. Next-Generation Sequencing

DNA studies have been revolutionized by the conventional Sanger sequencing technique, which was introduced in 1977 [32]. It is known as an enzymatic sequencing or chain-termination method, which utilizes labeled di-deoxynucleotides, acting as chain terminators [32]. The first human genome was sequenced based on Sanger sequencing technology in 2001, which took almost 13 years to complete and cost USD2.7 billion, and was part of a large collaborative and international publicly funded project [33]. In parallel, efforts to sequence the first human genome were also performed in a commercial setting by the company Celera Corporation, whose results were revealed in joint publications with the public Human Genome Project [34,35]. The Celera project employed a whole-genome shotgun sequencing approach and proceeded at a much faster pace and lower cost, although it benefited significantly from the data that was already generated by the public Human Genome Project [34,36]. As a result of both efforts to sequence the human genome, it became clear that the scale, efficiency, and cost needed to be vastly optimized for routine use in clinical diagnostics. Therefore, shortly after the release of the human genome sequence, the aim was re-established to achieve a USD1,000 human genome within 10 years [37].

Sanger sequencing is still routinely used for variant validation and has an extremely high accuracy of up to 99.999% [38]. However, it is considered a low-throughput technique, as up to one kilobase (kb) of DNA can be sequenced in 96 or 384 parallel reactions [39]. The technique has been optimized by the application of nucleotide-specific fluorescent labels and automated detection [40,41], the invention of polymerase chain reaction (PCR) [42], and the usage of polyacrylamide gels in capillary electrophoresis [41]. Therefore, DNA sequencing can be achieved within a shorter time frame and on a larger scale, in which the sequencing of millions of reads can be carried out in parallel, called “massive parallel sequencing” or “NGS”.

The NGS technique has rapidly overcome the limitations of traditional sequencing. Since 2005, various sequencing platforms, such as Illumina, Ion Torrent, Roche 454, and SOLiD sequencing, have been developed, which has resulted in a rapidly changing landscape during this new era of sequencing. The read length of these different platforms is shorter than that of Sanger sequencing (approximately 50–500 bp), with a higher error rate (0.1% in NGS compared to 0.001% in Sanger sequencing) [43]. However, the fast development of NGS techniques and the generation of public reference datasets containing population allele frequency data have allowed widespread integration of NGS technology in the research community and, later, in the clinical diagnostics of genetic diseases. Nevertheless, as whole-genome sequencing (WGS) is still relatively expensive and data interpretation is complex, a targeted sequencing approach (e.g., whole-exome sequencing (WES)), is often preferred.

#### 2.3.1. Targeted Capture Sequencing

Genomic regions of interest, such as the genes implicated in HL or RD, can be selectively enriched before sequencing is performed. There are various methods available to enrich target regions, such as hybridization-based, highly multiplexed PCR-based, and targeted circularization-based approaches. Extensive studies have been performed, which have applied these techniques to unravel genetic defects involved in inherited HL and RD. In 2013, Chio et al. investigated 32 cases with familial nonsyndromic HL, in which they reached a molecular diagnostic rate of 37% using a candidate gene sequencing approach of *GJB2*, *SLC26A4*, *POU3F4*, or mitochondrial genes based on observed clinical features and inheritance patterns. Later, by application of hybridization-based target capture sequencing for 80 HL-associated genes, they were able to increase the total diagnostic detection rate to 78% in this cohort [44]. In 2017, Dockery et al. utilized the hybridization-based enrichment method to sequence 254 IRD-associated genes in over 750 affected individuals in Ireland, in which they could identify pathogenic variants in 68% of the cases [45]. A recent study by Khan et al. applied a highly multiplexed PCR-based approach, with single-molecule molecular inversion probes (smMIPs), to sequence the complete *ABCA4* gene (coding and noncoding regions) in 1054 individuals with Stargardt disease (OMIM: 248200), who were previously screened for variants in the coding regions and remained genetically unexplained. This study proved that a smMIP-based approach is a cost-effective approach in the case of a strong genotype–phenotype correlation. The method allowed deep-sequencing of the region of interest, and causal structural and deep-intronic variants were identified in 25% of the investigated cases who were genetically undiagnosed after prescreening methods [46].

Targeted NGS techniques have several advantages, such as less data storage, high sequencing accuracy due to high coverage, and cost- and time effectivity [47]. However, this approach is unable to detect variants in novel (candidate) disease-associated genes. Furthermore, pathogenic variants residing in noncoding regions and structural variants (SVs) can be missed if only exons are analyzed. Due to decreasing prices of both WES and WGS, these approaches have become rapidly preferred to overcome the disadvantages of targeted NGS.

#### 2.3.2. Whole-Exome Sequencing Versus Whole-Genome Sequencing

Protein coding regions comprise 1–2% of the human genome. However, it is estimated that they harbor approximately 85% of disease-causing variants [48,49,50]. Therefore, the enrichment of coding regions utilized in WES quickly became an accurate and efficient method to investigate the coding regions of the genome for potential pathogenic variants, and this is now widely applied in genetic diagnostics [51]. One of the striking features of WES is in the success rates of genetic diagnostics of diseases with extensive locus heterogeneity, such as inherited HL and RD [9,52]. Currently, the diagnostic yield for RD using WES is estimated to be between 50% and 80%, dependent on the phenotype studied [9,53,54,55]. According to a study performed by Haer-Wigman et al., the highest yields were obtained for retinitis pigmentosa (63%), and the lowest yields were obtained for macular dystrophy (28%) and rare unspecified types of RD (25%) [9]. For HL, the genetic diagnostic rate is also highly dependent on phenotype (e.g., syndromic or nonsyndromic phenotype, mode of inheritance). The highest rates are observed in patients with a positive family history or with a congenital or symmetric type of HL [52]. The overall estimated detection rate for HL, when employing WES, varies between 30–40% based on different large-cohort studies and largely depends on phenotypic diversity [8,56]. The diagnostic yield for HL is importantly influenced by the involvement of environmental factors (e.g., noise, ototoxic drugs, and trauma), which likely explains the difference in yield compared to RD. Genetic causes have been estimated to underlie approximately two-thirds of the cases of congenital and early childhood HL; the remaining cases can be explained by acquired causes [57]. This genetic contribution decreases with the patient’s age due to increased exposure to damaging environmental factors during life. In line with this observation, there are several reports of a negative correlation between diagnostic yield and age of onset of HL [8,52].

Despite the successes of WES in clinical settings, this technology is inaccurate in detecting SVs, such as a deletion of a single exon, and does not allow variant detection in deep-intronic regions or regulatory elements. Therefore, WGS may be preferred as it provides more evenly distributed and uniform read coverage and it is capable of detecting different types of variants across the entire genome [58,59,60,61]. In 2017, Carss et al. investigated a large cohort of RD patients, in which WGS was performed for 605 cases, WES for 72 cases, and, for 45 cases, both technologies were performed [61]. They identified disease-causing variants in 56% of all individuals (404/722), while, by using WES alone, the diagnostic yield was calculated to be 50%. Subsequently, 45/58 cases that remained unexplained by WES underwent WGS, and pathogenic variants were identified in 14 cases. The authors concluded that WGS has a great power to detect pathogenic SVs, variants in noncoding and regulatory regions, and variants in GC-rich regions. The application of WGS revealed the pathogenic variants in 31% of the cases that remained unsolved after WES. These variants were missed mainly due to the poor quality of reads or the incapability of WES to identify SVs [61]. The prices for WGS keep decreasing [62], and the importance of the noncoding regions of the genome has become more evident. Therefore, a shift from exome to genome sequencing will be observed in clinical diagnostics in the near future to overcome the diagnostic gap observed in the application of WES. In 2020, Méjécase et al. provided a practical and cost-effective guideline for current and future genetic testing of RDs, in which they proposed to utilize WES or targeted NGS for the initial screening of exons and flanking intronic regions of (candidate or known RD) genes, reserving WGS solely for cases that remained unresolved [63].

Although NGS techniques have revolutionized the field of medical genetics, these short-read sequencing (SRS) approaches pose several limitations, such as (1) difficulties in the identification of complex and large SVs, (2) inability to sequence repetitive regions, (3) the lack of phasing of alleles, and (4) difficulties in distinguishing highly homologous regions such as pseudogenes [64]. These limitations may play a significant role in the diagnostic gap in medical genetics.

### 2.4. Third-Generation Sequencing

Due to the limitations of the aforementioned NGS techniques, there has been a need to develop new sequencing approaches to overcome these issues. The era of third-generation sequencing arrived in 2011 when Pacific Biosciences (PacBio) released a novel sequencing technique called “single-molecule real-time” (SMRT) sequencing [65]; only three years later, Oxford Nanopore technologies introduced nanopore sequencing [66]. Although these two techniques utilize different approaches to sequence genomic DNA, they share two major advantages compared to NGS. First, they are established on PCR-free and real-time sequencing processes, and second, they generate ultra-long sequencing reads, >10 kb [64,67]. These long-read sequencing (LRS) technologies are revolutionizing the genetics field as they provide a further understanding of the normal and morbid anatomy of the human genome and can, thereby, fill the gaps in the molecular diagnostics of genetic diseases.

#### 2.4.1. Single-Molecule Real-Time (SMRT) Sequencing

**Figure 1:**
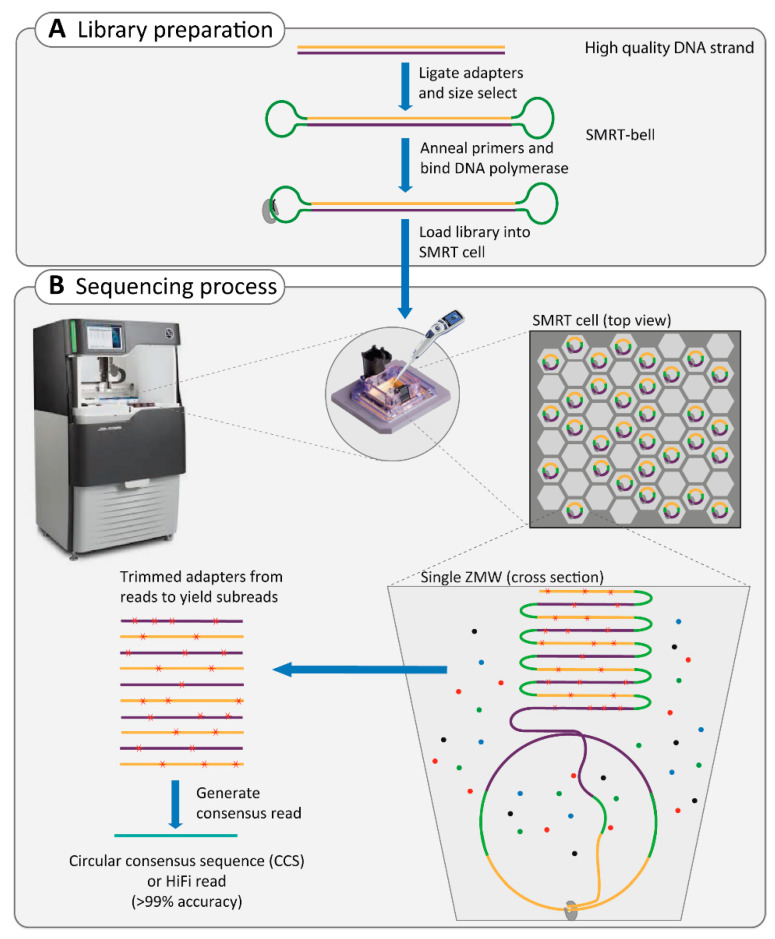
Overview of single-molecule real-time (SMRT) sequencing technology. (**A**) Sequencing starts with library preparation of ultra-long double-stranded DNA. In the next step, adapters, DNA polymerase, and primers bind to the double-stranded DNA, creating the SMRT-bell, which will be loaded later to the SMRT-cell. (**B**) The library is randomly distributed in the SMRT-cell in the sequencer instrument, in the ideal condition one-third of the ZMWs will be loaded by an SMRT-bell. In each ZMW, the DNA polymerase together with an SMRT-bell are bound to the bottom of the ZMW. The SMRT sequencing uses the circular DNA template to generate a continuous long read in each ZMW chamber. Afterwards, the adapters are trimmed from this long read and overlapping reads can be combined to one consensus sequence of high quality called HiFi read.

SMRT sequencing relies on ligating hairpin adapters to both ends of the double-stranded template DNA molecule (dsDNA), thereby circulating the dsDNA into the construct called the SMRT-bell. In the next step, primers and DNA polymerase are annealed to the adaptor in the SMRT-bell, which will later be utilized for circular consensus sequencing (CCS; Box 1, Figure 1A). The CCS approach can obtain approximately 83% accuracy (10× coverage, on average), with a 13–15% error rate dominated by small insertions and deletions [67,68]. This can be improved to 99% accuracy by selectively sequencing a targeted region with increased coverage of 15× [69,70,71]. SMRT technology is a PCR-free approach and requires minimal amounts of reagents and a simple library preparation procedure by which ultra-long dsDNA can be obtained. This technology can provide the result within hours, compared to several days for previous approaches [65]. An average read length of 10–15 kb can be reached, which allows de novo assembly, phasing of variants and haplotyping, and the detection of large SVs throughout the genome [64,72,73].

Box 1 **Single-molecule real-time (SMRT) sequencing technique.**

To enable the sequencing of single DNA molecules in real-time, two obstacles had to be overcome. First, concentrating the DNA polymerase and its template, the SMRT-bell (Figure 1A), to the very small observation chambers, which creates a higher signal-to-noise ratio. This problem has been solved by zero-mode waveguide (ZMW) technology, a small hole of approximately 45 nanometers (nm) in diameter [74]. The DNA polymerase, with its template, is anchored by a strong biotin/streptavidin interaction to the bottom of the ZMW. Therefore, the laser illumination of incorporating nucleotides is limited to the bottom, which increases the signal-to-noise ratio [68], as ZMW can efficiently distinguish signals of nucleotide incorporation against the background of unincorporated nucleotides (Figure 1B).

The second obstacle in real-time sequencing of single DNA molecules was the large size of the fluorescent dye, which interfered with the normal activity of DNA polymerase and caused the halting of the enzyme shortly after the initiation of DNA synthesis. In SMRT technology, the dye is attached to the phosphate chain instead of the nucleotide, which is naturally cleaved during DNA synthesis after nucleotide incorporation; this results in a single long, natural DNA strand [68].

The real-time sequencing of the circular SMRT-bell is performed in each ZMW, which generates continuous long reads (Figure 1B). During data processing, the adaptors are removed, and subreads are generated. Subsequently, the combined subreads enable the generation of one highly accurate consensus sequence called the circular consensus sequence (CCS).

#### 2.4.2. Nanopore Sequencing

**Figure 2:**
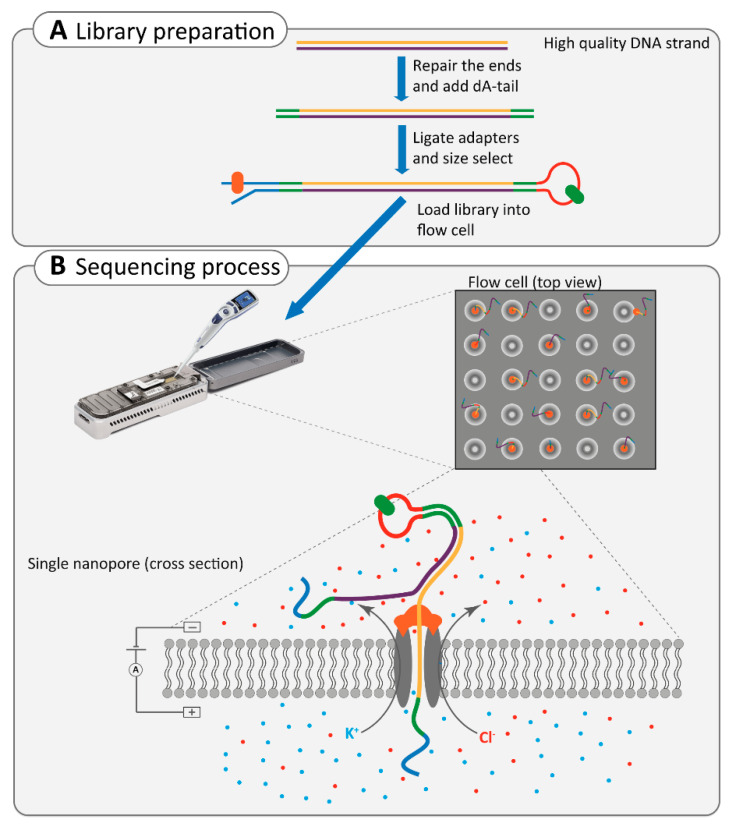
Overview of the Nanopore sequencing technology. (**A**) The library preparation includes end-repairing, and adding dA-tails, followed by ligation of two types of adapters to both ends of the ultra-long double-stranded DNA. The adapters carry the motor enzyme (in orange) and hairpin-protein (in green) to facilitate the movement of DNA through the nanopore and ensure the sequencing of the second strand of DNA, respectively. (**B**) The library is loaded into the flow cell in the sequencer instrument. The flow cell contains thousands of nanopores that allow the flow of Cl^−^ and K^+^ ions between two compartments. The motor enzyme anchors to the nanopore and unwinds the DNA to pass it through the pore. Thereby, the electric current is influenced based on the unique shape of each nucleotide in single-strand DNA. These changes in the electric current are later translated to sequences.

Nanopore sequencing is an advanced third-generation sequencing technique that offers straightforward sample preparation, requiring minimal reagents or amplification processes [75]. This technology relies on transferring a DNA molecule through a pore and directly detecting each nucleotide by its effect on an electric current (Box 2, Figure 2) [76,77].

Box 2 **Nanopore sequencing technique.**

Nanopore sequencing occurs in a flow cell, in which two ionic solution compartments are separated by a membrane containing thousands of nanopores. The flow of electric current between these two compartments depends on the molecule transferring through one of the pores. Since each nucleotide differs in shape, its effect on the electric current is specific for each nucleotide (Figure 2) [67,77,78]. Library preparation in nanopore sequencing includes the end-repair of ultra-long dsDNA, the addition of dA-tails, followed by the size selection and ligation of adapters, which are protein-DNA molecules. The first adapter is the leader-adapter, which contains a motor enzyme. It binds to the nanopore and ensures the gradual movement of DNA through the pore. The dsDNA is then unwound at the pore, and only one strand will pass through the nanopore. The second adapter is a hairpin-adapter containing a hairpin protein. It generates one long single strand of DNA, which ensures the sequencing of the second strand of DNA in order to increase the accuracy of sequencing [66,67,79].

There is no limit in the length of DNA that can be sequenced with this technique since it does not require DNA amplification or synthesis. However, the challenge lies in library preparation, which needs to result in ultra-long dsDNA molecules [80]. The aver-age size of reads is usually above 10 kb, and for some ultra-long dsDNA molecules, it can reach one Mb [64]. The main drawback of nanopore sequencing technology is its relatively high error rate of ~20%. Compared to SMRT technology, in which the error rate can be re-duced by high coverage due to CCS, in nanopore sequencing, it is a systematic error, and correction can only be achieved by comparison to short-read sequence data [80]. Never-theless, this technology is rapidly improving to overcome the current issues (such as error correction), and library preparation is being optimized to achieve high-quality ultra-long dsDNA [77].

### 2.5. Application of Third-Generation Sequencing in Inherited HL and RD

Third-generation sequencing has revolutionized the field of medical genetics by its superior performance in the analysis of repeated and highly homologous regions, SVs, haplotype phasing, and transcriptome analysis [81]. These technologies are currently mainly used in research applications and show great promise to overcome the disadvantages of SRS methods. In a systematic analysis, Ebbert et al. compared the performance of whole-genome SRS and LRS technologies at repetitive regions in the human genome. Amongst others, they showed that 8.6% of the protein-coding regions of *RPGR* (associated with X-linked RD) and 12.7% of the protein-coding regions of *OTOA* (associated with HL) are within the unmapped reads of SRS data, which were resolved by performing LRS. Specifically, they indicated that nanopore sequencing outperforms PacBio sequencing by resolving 90.4% and 64.4% of the SRS-unmapped regions, respectively [82].

One important application has been to identify complex SVs associated with genetic diseases, including HL and RD. Reiner et al. utilized SMRT LRS to detect a 72.8-kb deletion region in the *BBS9* gene and map the breakpoints at the nucleotide level in a patient diagnosed with Bardet–Biedl syndrome (OMIM: 615986). This deletion was determined to be the causal variant and a founder mutation in the Guyanese population [83].

In another recent study, researchers utilized transcriptome sequencing, followed by short- and long-read WGS, to identify a 7.4-kb duplication in *NMNAT1*, which spans two out of five exons of this gene. The duplication caused a previously unrecognized autosomal recessive syndrome, symptoms of which are Leber congenital amaurosis and sensorineural HL, which occur together with other features such as spondyloepiphyseal dysplasia, intellectual disability, and brain anomalies. The authors were able to determine the exact breakpoints of the duplicated region, missed by previous approaches, as well as two *Alu* elements flanking this segment, which are potentially involved in the origin of the SV [84].

Recently, nanopore sequencing enabled researchers to unravel the genetic defect in two unrelated patients diagnosed with mild-to-moderate HL. Nanopore sequencing revealed a gene conversion event between the *OTOA* gene and its pseudogene, in which exons 21 to 23 of *OTOA* were replaced by exons 1 to 3 of *OTOAP1* [85]. As pathogenic variants within the *OTOA* gene have been described to cause autosomal recessive HL (DFNB22; OMIM: 607039), this gene conversion event was considered causative [85].

Despite the advantages of LRS techniques, they possess multiple important drawbacks that prevent a wide range of uses outside research applications. One of these is the relatively high costs compared to SRS NGS technologies (USD800–2000 per run, depending on the different platforms and instruments), based on the lowest possible flow cell price and highest output [67]. The other major disadvantage is the requirement for high-quality ultra-long dsDNA, which can be challenging to obtain. In particular, for nanopore sequencing, the required fresh blood samples for DNA extraction can also be a hurdle. However, as LRS technologies are rapidly decreasing in price and are continuously improving in different aspects, such as optimized library preparation and error correction, it is expected that these technologies will eventually enter routine genetic diagnostics in Western countries. In addition, like SRS, targeted LRS can also be performed by targeted amplicon sequencing, CRISPR/Cas-based targeted enrichment, or using a “Read Until” approach in order to enrich for genetic loci associated with a specific phenotype. Targeted LRS is a cost-effective and efficient strategy to investigate high-priority loci in unsolved cases [86,87]. For both HL and RD, several associated genetic loci (44 and 36 loci, respectively) have been described, for which the implicated genetic defect is still elusive [1,2], and, therefore, a targeted LRS approach could be of interest.

**Figure 3:**
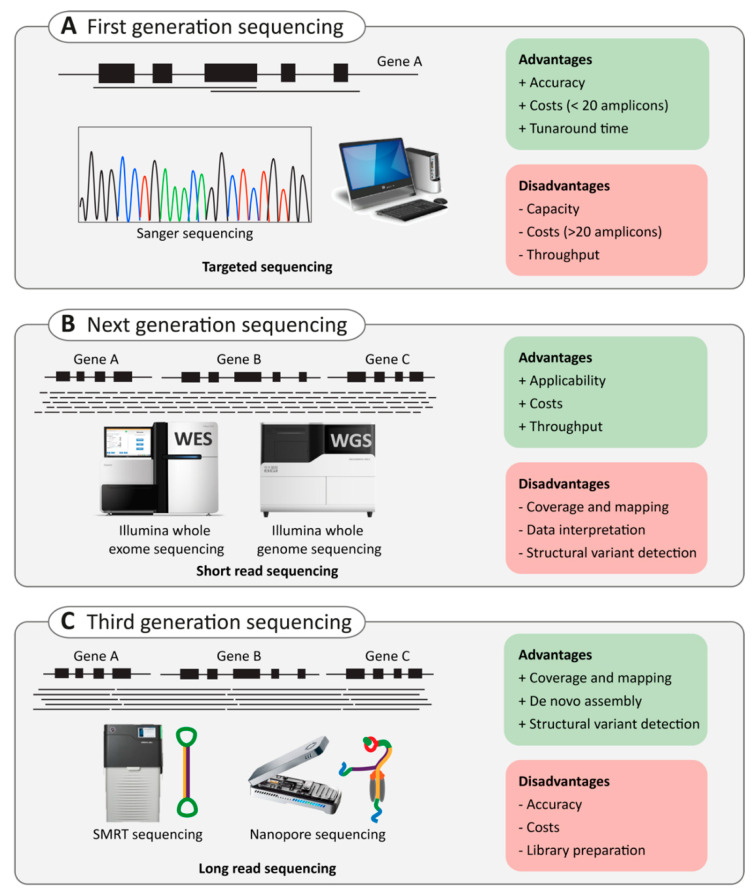
Comparison of conventional Sanger, next-generation, and third-generation sequencing. (**A**) schematic representation of (**A**)) first generation sequencing (Sanger sequencing), (**B**) next generation sequencing (e.g. Illumina whole-genome sequencing (WGS) and whole-exome sequencing (WES)) and (**C**) third-generation sequencing (e.g. SMRT sequencing as performed by Pacific Biosciences (PacBio) and nanopore sequencing by Oxford Nanopore Technologies (ONT)). For each technique, advantages (green) and disadvantages (red) are provided.

Finally, as sequencing technologies develop and improve rapidly (Figure 3), the next challenge will lie in bioinformatics, data storage, data analysis, and variant interpretation of NGS or LRS data. A high number of different variants are revealed by these methods. However, not all these variants are disease-causing. Therefore, special attention is being paid to prioritization processes that can aid in decreasing the number of putative candidate variants. In addition, developments in bioinformatic tools are needed to better interpret the effect of candidate variants. In the next section, we will discuss the importance and challenges of variant interpretation and the importance of this matter in clinical application.

## 3. Variant Interpretation

The total length of human DNA is over 3 billion base pairs, and it holds, on average, 4–5 million variants compared to the healthy human reference genome, which highlights the obvious challenge of distinguishing potential disease-causing variants from benign variants or polymorphisms [88]. For protein-truncating variants, a potential pathogenic consequence is often evident, while missense, synonymous and noncoding variants are more challenging to interpret. Moreover, with increased knowledge regarding the involvement of noncoding DNA in human disease development, the complexity of data to be analyzed has gone through the roof.

In 2015, the American College of Medical Genetics (ACMG) provided a framework to utilize and standardize sequence variant interpretation for Mendelian disorders [89]. Each variant is categorized using a uniform scoring system: benign, likely benign, uncertain significance, likely pathogenic, or pathogenic. The classification system employs several hierarchical steps, which include the use of literature and databases, computational and predictive data, functional data, and segregation analysis. Variant classification is the cornerstone of clinical molecular genetic testing. Therefore, ACMG guidelines provide a consistent and well-applicable system to guide this process. On the other hand, for research focused on the identification of novel gene-disease associations, the ACMG guidelines are more difficult to apply and less suitable.

### 3.1. Literature and Database Use

Variant frequency databases are a useful resource for allele frequencies of variants in large populations. As a rule of thumb, the frequency of a disease-causing variant should not be higher than expected, based on the incidence or prevalence of the genetic disorder [90]. The most comprehensive allele frequency database today is gnomAD (successor of ExAC), which contains frequency data for both SNVs and SVs based on 91,864 genomes and 125,748 exomes [91]. Additionally, this database provides variant frequencies for many subpopulations, which allows the usage of population-matched control data. Nevertheless, some populations (e.g., African/African–American) remain underrepresented, which limits efforts in precision and personalized medicine for these ethnicities. Several efforts are ongoing to obtain more (high-quality) genomes from these populations [92,93]. Databases such as gnomAD [91], goNL [94], UK10K [95], and Wellderly [96] contain sequencing data of (presumably) healthy cohorts. However, important caveats related to age-of-onset and reduced penetrance should not be ignored.

Unlike population databases, disease databases such as ClinGen [97], ClinVar [98], Leiden Open (source) Variation Databases (LOVDs) [99], and the Human Gene Mutation Database (HGMD) [100] also provide genotype–phenotype information. All the variants collected in the HGMD database have been reported in patients and likely disease-causing. They have been published in the literature and manually curated. The Deafness Variation Database (DVD) provides a comprehensive catalog for genetic variation in genes associated with HL [101]. Efforts are ongoing to collect and annotate all published variants associated with inherited nonsyndromic RDs, Bardet–Biedl syndrome, and Usher syndrome into 195 gene-specific LOVDs [28,102,103,104,105,106].

Several studies have proven the value of incorporating population frequency data as a variant prioritization strategy and have successfully clarified variants of unknown significance [61,107]. However, an important caveat is that a reliable database should be frequently updated, and uploaded sequencing data should adhere to quality control criteria. An example of non-pathogenic variants mistakenly reported as pathogenic has been highlighted in a study performed by Hanany et al. [108]. The authors extracted up-to-date allele frequencies from gnomAD of variants in genes associated with dominantly inherited RD and concluded that the pathogenicity of variants in 19% of these genes should be debated. Inherited HL, on the other hand, is a more common condition, than RD and therefore the expected maximum allele frequency for a pathogenic variant should be adjusted accordingly [109].

Once a potentially disease-causing variant is identified, a rich source of available scientific and medical literature can be assessed. A first important step entails thorough comparisons between the observed phenotype in the investigated proband and phenotypic observations described in the literature. Most well-described phenotype–genotype correlations can be found in data repositories: Online Mendelian Inheritance in Man (OMIM) [110], ClinGen [97], and ClinVar [98].

Strong phenotype–genotype correlations are complicated by a phenomenon called allelism—different phenotypes can result from different alleles of the same gene [111]. For example, autosomal recessive Stargardt disease (STGD1), which is due to two variants or alleles in *ABCA4*, shows a wide clinical spectrum of maculopathies [112]. The most severe form is early-onset (onset <10 years) STGD1 or panretinal cone-rod dystrophy, which is due to two deleterious *ABCA4* alleles. Classical or intermediate STGD1 (onset between 10 and 40 years) is due to a combination of a deleterious variant and a mild variant. Finally, late-onset STGD1 (onset >40 years) is caused by a deleterious variant and a mild variant (p.Asn1868Ile), showing reduced penetrance [112,113,114]. Truncating variants in the *CDH23* gene are assumed to cause Usher syndrome type 1D (OMIM: 601067), which consists of HL and retinitis pigmentosa, whereas missense variants cause nonsyndromic HL (OMIM: 605516) [115]. However, several exceptions to this rule have been reported [116,117]. For pathogenic variants in the *USH2A* gene that can cause both nonsyndromic retinitis pigmentosa and Usher syndrome type IIa (OMIM: 276901), the correlation of missense and truncating variants with the associated phenotypic expression is not always clear, although truncating *USH2A* variants are more frequently reported in patients diagnosed with a syndromic phenotype [118,119]. Additionally, variants affecting genes that are implicated in ciliopathies (e.g., *BBS1, CEP290*, *IQCB1*) can cause a wide range of variable symptoms that are part of a (syndromic) phenotype. Symptoms described for ciliopathies often include retinal degeneration and, less frequently, HL (reviewed in [14]). To date, >80 forms of syndromic RD have been described, which are linked to approximately 200 IRD-associated genes [120]; for syndromic HL, these numbers are suggested to be even higher [121].

Besides a phenotypic resemblance, the expected mode of inheritance and the involved pathogenic mechanism of the variant (e.g., haploinsufficiency, loss- or gain-of-function) should also be compared with literature reports. For genes that have not been previously associated with the disease of interest, OMIM [110] and GeneCards [122] provide a summary of known clinical and functional information for the gene. For candidate disease genes, it may be valuable to investigate gene expression in the tissue of interest (e.g., SHIELD [123], gEAR [124], EyeGEx [125]) and explore associated protein interaction networks (e.g., STRING [126]). Additionally, the initiative Genematcher [127] and the European Retinal Disease Consortium (ERDC) [128] offer the opportunity for different research groups that share an interest in the same candidate disease gene to collaborate. It is hypothesized that the most prominent genetic causes of diseases have been identified, and novel findings appear in few cases or families, which underlines the urgency for collaborations among research groups worldwide. It is of utmost importance to share candidate disease gene data to increase the likelihood of identifying multiple unrelated individuals affected by pathogenic variants in the same gene [129,130].

### 3.2. Computational and Predictive Data

The spectrum of human genetic variation is diverse, and a rich source of bioinformatics tools has been developed to evaluate the different potential consequences of a variant. Although the pathogenicity of SNVs has been most extensively studied, recent efforts into the characterization of SVs have revealed that pathogenic SVs are more abundant than initially thought [90,131]. This has led to a gradual shift of attention from coding variations to structural variations and the noncoding regions of the genome.

#### 3.2.1. Null Variants

Null variants are considered very strong evidence of pathogenicity and often lead to open reading frame disruption and, consequently, the complete loss of protein function. Null variants include nonsense, frameshift, canonical splice site, and initiation codon variants, as well as out-of-frame single- and multiexon deletions. Available in silico prediction tools are often not designed for the interpretation of null variants, as pathogenicity already seems evident in most cases. However, some caveats should be considered, including the presence of alternative transcripts, the position of the variant with respect to 3’UTR, and the inducement of alternative splicing such as in-frame exon skipping as a putative correction mechanism [132,133,134]. For each gene, a loss-of-function intolerance (pLI) score, which is based on observed (homozygous) loss-of-function variants in healthy cohorts compared to the expected number based on the gene size, is provided in gnomAD [91].

#### 3.2.2. Missense, Synonymous, Indel, and Intronic Variants

Substitution variants located in the coding (exonic) or noncoding (intronic) regions of a gene are more difficult to interpret. Missense variants and small in-frame insertions or deletions (indels) lead to changes in amino acid composition. Several computational tools have been developed to aid in the assessment of deleterious consequences of the identified variants. Output scores provided by these tools are usually based on evolutionary conservation of the altered nucleotide or amino acid residues, biochemical consequences of the amino acid change, or the location and context of the residue within the protein sequence, e.g., in a domain with a specific function. The most widely applied tools are combined annotation-dependent depletion (CADD) [135], Grantham [136], MutationTaster [137], PhyloP [138], PolyPhen-2 [139], and sorting intolerant from tolerant (SIFT) [140].

Alternatively, synonymous, missense, and (deep)-intronic variants can disrupt the normal splicing machinery and alter pre-mRNA processing. Variants can introduce or strengthen cryptic splice sites, disrupt canonical donor or acceptor splice sites, or disrupt the (binding) motifs that are essential for correct splicing processes, such as exonic splicing enhancers or silencers [107]. This can lead to alternative splicing events, such as pseudo-exon inclusion, exon elongation, or (partial) exon skipping. Potential splice-altering variants can be evaluated based on nucleotide conservation scores or by performing splicing assessments using predictive splicing algorithms, such as Human Splicing Finder [141], SpliceSiteFinder-like [142], MaxEntSCan [143], GeneSplicer [144], NNSPLICE [145], and SpliceAI, a deep learning algorithm [146]. In vitro midi- or minigene splice assays can be performed to confirm the predicted alternative splicing events in HEK293T cells or, if transcript levels allow, aberrant splicing can be detected in RNA derived from (EBV-transformed) blood cells [147,148].

One pitfall of splice site prediction tools is tissue-specific splicing of exons, which prevents most current prediction tools from detecting cochlear- or retina-specific splicing effects. Recently, Riepe et al. benchmarked different established and deep-learning tools on sets of variants in tissue-specific genes *ABCA4* and *MYBPC3* and observed that SpliceAI is the best performing splice prediction tool for both noncanonical splice sites and deep-intronic variants in *ABCA4* [149]. Moreover, Rowlands et al. compared seven machine and deep learning-based splice prediction tools and demonstrated that SpliceAI is superior in both sensitivity and specificity [150].

#### 3.2.3. Regulatory Variants

Variants located in intergenic and intronic regions of the genome can exert their pathogenic effect through a variety of mechanisms. Variation can occur within characterized cis-regulatory elements (CREs), such as promoters, enhancers, or insulators [151,152]. Regulatory elements are short DNA sequences (100–500 bp) that allow precise spatiotemporal control of gene expression levels [151]. Promoter and distant enhancer regions interact with each other via chromosomal looping, allowing the recruitment of transcriptional machinery. Alternatively, insulators can block the interactions between promoters and enhancers. An enhancer element can be located up to one Mb away from its target gene and can serve as the transcriptional regulator of one or more genes [151,153,154,155]. Usually, an enhancer displays a spatiotemporal pattern of activity. Transcription factors that bind enhancer or promoter elements are the key regulators of these processes, and they modulate gene expression. Pathogenic variants in cis-regulatory elements can alter transcription factor binding sites or the chromatin landscape and, therefore, the activity of the enhancer or promoter [151,152]. Databases such as JASPAR [156], which contain consensus sequences of transcription factor binding sites, can be used to predict the effect of a potential regulatory variant on transcription factor binding.

**Figure 4:**
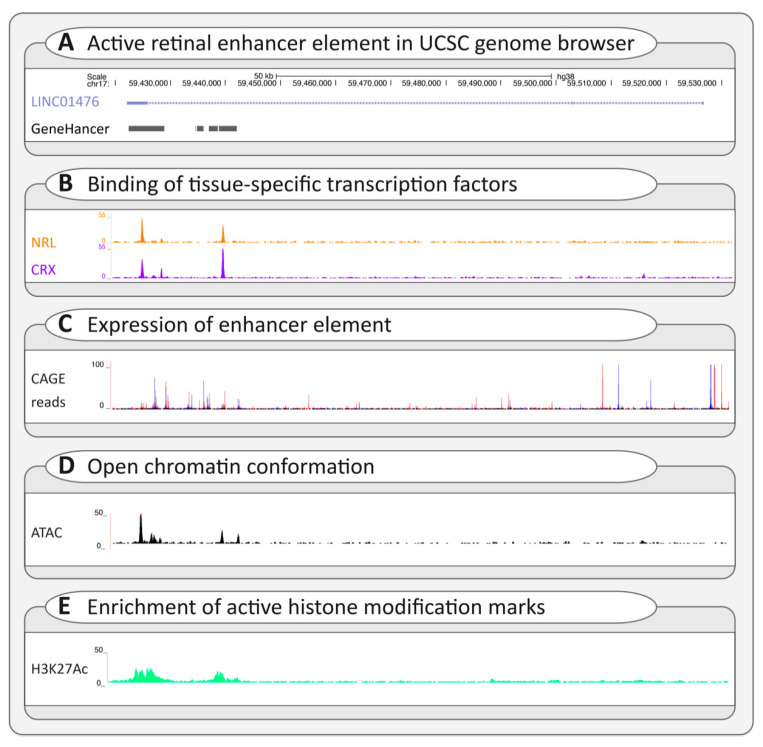
Interpretation of potential regulatory variants using the University of California Santa Cruz (UCSC) genome browser. To evaluate a potential regulatory variant, several publicly available datasets in the UCSC browser can be assessed to determine whether a variant is located within an active cis-regulatory element based on the presence of active enhancer or promoter hallmarks [162]. (**A**) A known active retinal enhancer element, described by de Bruijn et al. and included in the GeneHancer database, is visualized in the UCSC browser [159,161]. The enhancer element overlaps with a long noncoding gene, *LINC01476*, and is predicted to bind to the promoter region of the *YPEL2* gene (GeneHancer) [159]. The enhancer element is enriched for several active enhancer hallmarks. (**B**) Firstly, the element is bound by retina-specific transcription factors (NRL and CRX), as confirmed by ChIP-sequencing performed on a human retina sample (GEO database: GSE137311) [151]. (**C**) Secondly, cap analysis gene expression (CAGE) allows 5′ end sequencing of cDNAs, confirming the expression of the enhancer element, as shown by the FANTOM5 CAGE human dataset (Data available from https://fantom.gsc.riken.jp/data/) (accessed on March, 2021) [162]. (**D**) Thirdly, an open chromatin conformation of the enhancer element is confirmed by ATAC-sequencing of a human retina sample (GSE137311) [151]. (**E**) Lastly, the enhancer element is enriched for histone-modification marks that are associated with enhancer activity, such as H3K27Ac, as determined using ChIP-sequencing performed on human retina (GSE137311) [151].

Regulatory variants that impact transcription initiation usually lead to subtle changes in gene expression and are difficult to assess [152]. Therefore, context-specific profiling of the tissue- and cell-type-specific cis-regulatory architecture is essential [157]. Enhancer databases such as the ENCODE portal [158], GeneHancer [159], and EnhancerAtlas [160] contain an overview of reported cis-regulatory elements that are widespread throughout the genome. Potential interactions between promoter and enhancer elements can be assessed by evaluating available chromosome conformation capture data like Hi-C. Additionally, the presence of context-specific active enhancer hallmarks should be assessed. These include (1) the confirmed binding of transcription factors, (2) the production of noncoding enhancer RNA, (3) an open chromatin conformation, and (4) the presence of histone-modification marks that are associated with enhancer activity, such as histone 3 lysine 27 acetylation [151,157]. Figure 4 provides an overview of these hallmarks, the suitable techniques to assess these, and a selection of relevant publicly available (epigenetic) datasets used to interrogate the recently resolved autosomal dominant retinitis pigmentosa RP17 locus [161]. Once a candidate regulatory variant has been identified, experiments such as an in vitro luciferase reporter assay could be applied to confirm its effect on enhancer or promoter activity [151].

#### 3.2.4. Structural Variants

SVs are defined as genomic rearrangements that are larger than 50 bp [131]. SVs include deletions and duplications, also referred to as copy number variations, as well as inversions, translocations, and insertions [153]. In 2020, an amendment of the ACMG guidelines was published to aid in the classification of SVs [163]. SVs can have direct consequences on gene dosage levels when (partially) overlapping with coding regions of a gene or can cause changes in gene expression levels or patterns when overlapping with regulatory elements such as enhancers. Additionally, SVs that are limited to the noncoding regions of the DNA can interfere with the 3D genome structure and disrupt cis-regulatory architecture [131]. Each chromosome is compartmentalized in regulatory units, so-called topologically associating domains (TADs). Within each TAD, enhancers and gene promoters can interact. Neighboring TADs are shielded from each other by boundaries, which are typically occupied by the transcription factor CTCF [164]. Disruption of TAD architecture by SVs can have severe pathogenic consequences. Deletions can lead to the fusion of neighboring TADs, inversions can result in the exchange of regulatory sequences, and duplications can generate novel TAD compartments, leading to ectopic enhancer-promoter contacts (neo-TADs) [153,165,166,167]. These genomic rearrangements can result in pathogenic alterations of gene expression levels. Recently, it was shown that TAD rearrangements caused by SVs are an important cause of autosomal dominant retinitis pigmentosa (RP17) [161]. Additional studies, focused on the identification of copy number variants involved in HL or RD, have also suggested a prominent role for pathogenic SVs [168,169]. To predict the potential consequences of structural rearrangements, the epigenetic landscape of the region, including the presence of CTCF sites, interactions, and directionality, should be evaluated.

### 3.3. Segregation Analysis

Once a candidate disease-causing variant is identified, segregation analysis should be performed, if possible, to confirm that the observed inheritance of the variant matches the family history. If a variant is segregating with the phenotype within the family, this could serve as supportive evidence for linkage of the identified variant to the disorder. However, the variant might still be in linkage equilibrium with the true pathogenic variant, and the genetic locus should always be carefully screened for missed variants. Additionally, a careful clinical evaluation of all family members is essential to exclude mild symptoms of reportedly unaffected individuals as well as possible phenocopies, whose phenotype can be explained by other (nongenetic) factors. The latter is especially relevant for cases diagnosed with inherited HL, as both genetic and environmental factors are significant contributors to the development of HL [3].

Other factors that might complicate the interpretation of segregation analysis results are age-related or reduced penetrance, modifiers, carrier females in X-linked diseases, and multigenic inheritance. Several studies have indicated that modifying variants can have higher allele frequencies than fully penetrant alleles and, therefore, are not recognized by diagnostic pipelines [170,171]. Despite their high allele frequencies, it has been shown that these variants can still significantly modify Mendelian genotypes. For instance, the variants p.(Ser192Tyr) and p.(Arg402Gln) in *TYR* have an individual allele frequency of 36.4% and 27.3% in the gnomAD database (non-Finnish Europeans), respectively, while the p.[Ser192Tyr;Arg402Gln] allele has an allele frequency of 1.9%. Despite the relatively high population frequency, the pathogenicity of the p.[Ser192Tyr;Arg402Gln] allele has been suggested when present in a homozygous state or in a triallelic genotype with a known pathogenic *TYR* variant in trans [172,173]. Studies suggest that one in six genes implicated in RD is possibly associated with variable penetrance due to variability in expression levels [174,175]. Examples of strong evidence for variants with reduced penetrance, implicated in RD or HL, have been reported for *ABCA4* [113,176], *COCH* [177], *PRPF31* [178], and *RIPOR2* [179].

Another complicating factor is uniparental disomy (UPD), where two homologous chromosomes are inherited from the same parent due to errors during meiosis. In 2020, Yauy et al. investigated the presence of UPD in exome sequencing data of 4912 trios [180]. The authors detected UDPs in 0.05–0.2% of these trios, amongst which was a chromosome 1 UPD (*ABCA4*) in a Stargardt disease case, suggesting minimal contribution to the genetic diagnostic yield [180]. Thus far, there are four reported Stargardt disease cases showing UPD in chromosome 1 [46,180,181,182]. Moreover, in 2013, Roosing et al. described maternal UPD of chromosome 6, which included a pathogenic *TULP1* variant responsible for the cone dystrophy phenotype of the proband. For HL, several cases of UPD have been described as well, affecting chromosome 1 (*USH2A*) [183], chromosome 13 (*GJB2)* [182], and chromosome 18 (*LOXHD1*) [182,184].

### 3.4. Functional Evaluation of Variants

Functional assays can provide an extra line of evidence that can aid in the discrimination between (likely) pathogenic variants, (likely) benign variants, or variants with unknown significance. For proteins with a well-characterized subcellular localization or function, in vitro approaches can be considered to assess the impact of the variant on protein localization or function. Examples of the latter are assessments of transporter function, enzymatic activity, or activity of metabolic pathways. In vivo experiments are ideal for studying the true biological context. However, as it is not always feasible to perform such studies, in vitro research can, instead, provide valuable information. Biochemical data obtained from patient-derived biopsies might be more informative. However, for both HL and RD, samples derived from the tissues of interest are usually not available. For these purposes, animal models could provide a valuable alternative. Over the years, several studies have proven the suitability of studying ear- or eye-related disease in nonhuman primates and mouse models [38,185]. The International Mouse Phenotyping Consortium (IMPC) aims to generate mouse knockout models for all known genes in the mouse genome [186]. Furthermore, the zebrafish has proven its suitability as an animal model. In this model, retinal and inner ear function can already be studied five days postfertilization [185,187]. Limitations in the usage of animal models include ethical, time, and financial considerations, in addition to the level of gene conservation.

Stem cell technology and the development of differentiation protocols over the past decades have enabled the in vitro generation of patient-derived cells, resembling retinal photoreceptors or inner ear hair cells [188,189]. These models can provide an alternative method of studying the tissue of interest. Research has shown that differentiated cells can resemble the patient’s retina or inner ear. Several 2D- and 3D-differentiation protocols have been successfully applied to study both HL and RD. Differentiation approaches are rapidly being optimized, as the involved processes are still very time-consuming and expensive [188,189]. More so, variability and cell heterogeneity are important hurdles, and these should be overcome in order to fully replace animal model studies.

## 4. Future Developments

### 4.1. Development of New Technologies

Chromosomal abnormalities and SVs are among the main causes of genetic diseases, which are being addressed in clinical application using routine cytogenetics methods such as karyotyping and fluorescent in-situ hybridization (FISH), comparative genomic hybridization (CGH), and SNP microarrays [190,191]. However, these methods manifest significant limitations in the identification of SVs. For example, karyotyping allows the identification of different chromosomal abnormalities with a 5–10 Mb resolution. Although microarrays and CGH arrays are able to identify the gain and loss of chromosomal material as small as 10 kb, balanced rearrangements cannot be detected by these methods nor the exact location of the structural variation [192,193]. It is estimated that only 15–20% of chromosomal abnormalities can be detected by the application of these techniques, which indicates the great need for new technologies in the field of cytogenetics [194].

**Figure 5:**
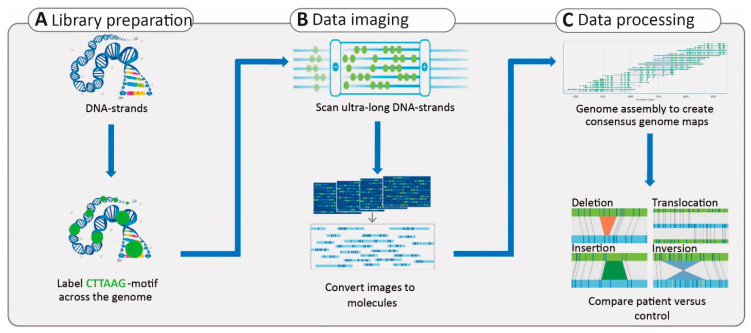
Overview of optical mapping technology. (**A**) High-quality DNA is isolated and later labeled at a 6-mer motif across the genome. (**B**) The labeled DNA is linearized in order to take images of the label patterns in the DNA molecules, and, subsequently, the images are converted to the molecules. (**C**) These molecules are then utilized for genome assembly to generate consensus genome maps. The pattern of labels can be compared between the reference genome and the affected individuals to identify structural variants. The shorter or longer distance between two labels indicates deletion or insertion, respectively. Translocations can be identified by the mapping of a single region in the patient genome to two genomic regions in the reference. The inverted pattern of labels in a patient, compared to that in the reference genome, indicates the presence of an inversion.

Although LRS techniques are rapidly developing and show a great ability to identify SVs, their routine application in clinical diagnostics still requires several improvements in terms of sequencing and variant interpretation; it also requires a cost reduction. In addition, despite the fact that these technologies can provide substantial read length, the reads can only be assembled to the scaffold level and not to the chromosome level [195]. Complementary approaches to identify SVs can be offered by cytogenetics [193]. One of these recent technologies is optical mapping (Bionano Genomics), which is a de novo assembly-based method that allows the visualization of the genomic structure in high resolution [196]. The approach is based on ultra-long dsDNA molecules that are fluorescently labeled at CTTAAG hexanucleotide motifs, which are found, on average, 15 times per 100 kb across the human genome. The distances and patterns of these labels can be compared to those in a reference genome. Therefore, copy number aberrations and other SVs, including insertions, inversions, and translocations, can be detected (Figure 5).

Optical mapping has a much higher resolution compared to standard karyotyping and microarray technologies and, therefore, enables much more precise data analysis. As it is an imaging method and not a sequencing method, SNVs cannot be detected. However, for the analysis of SVs, optical mapping can be used in a complementary manner to sequencing techniques [193]. With the ability to map ultra-long dsDNA molecules at a low cost, optical mapping has facilitated SV detection, haplotype phasing, and genome assembly [195]. In a recent study, researchers utilized optical mapping to identify a 48-kb duplication at the *LAMA1* locus that causes Poretti–Boltshauser syndrome (OMIM: 615960). Affected individuals present with ataxia, cognitive impairment, and language delay, as well as ocular phenotypes such as ocular motor apraxia, abnormal eye movement, and RD. WES and chromosome microarray prescreening methods failed to reveal the large SV in the studied family [197]. The authors reasoned that LRS technologies offer promising applications in comprehensive SV analysis; however, the costs and accuracy may represent a burden. Therefore, they suggested that a combination of different technologies, such as optical mapping and SRS, provides a more comprehensive understanding of SVs when considering cost, time, and throughput [197].

### 4.2. Multiomic Approaches

Besides genome sequencing, other omic technologies, such as transcriptomics, proteomics, metabolomics, or epigenomics, hold the promise to further close the diagnostic gap for RD and HL. It is evident that for each identified disease-associated gene, the isoform landscape and levels of involved gene regulation are more complex than initially thought. A quantitative (gene expression levels) or qualitative (isoform structures, novel exons) analysis of the transcriptomic landscape is valuable in enhancing diagnostic yield, as shown by several studies [198,199]. In combination with genome sequencing, RNA sequencing can improve the interpretation of variants with unknown significance, although inaccessibility of cell types for RD- and HL-associated genes is a major limitation.

LRS offers the potential for RNA analysis as well: for example, the Iso-Seq method of PacBio enables the sequencing of full transcripts, and nanopore sequencing offers direct sequencing of RNA molecules [64]. LRS techniques have already shown to be successful in the identification of novel full-length transcripts. In a study performed by Ray et al., an abundant retina-specific *CRB1* transcript (*CRB1-B*) was detected, which was not annotated in genome databases such as the UCSC genome browser [200,201]. The authors showed that the expression of the *CRB1-B* transcript is significantly higher in photoreceptors than the canonical *CRB1* transcript (*CRB1-A*). The newly identified transcript includes unique exons that are not present in *CRB1-A* and, thereby, represent important candidate regions for potentially missed pathogenic variants [201]. In addition, developments in the single-cell RNA sequencing field allow the identification and characterization of tissue-specific isoforms and regulatory events. The Single Cell Portal (Broad Institute) offers a valuable resource of tissue-specific single-cell RNA sequencing datasets [202].

Epigenomics is an emerging and promising development in the field of medical genetics. Analysis of epigenomic signatures can aid in the understanding of the 3D organization of the genome. Since base modifications remain captured in native DNA molecules that are used for SMRT and nanopore sequencing, investigation of the methylome and DNA base modification is possible [64,67]. Ideally, multiomic layers (e.g., genomics, transcriptomics, and epigenomics) should be integrated (the so-called multiomics), which aids in an ultimate understanding of the genomic landscape and provides valuable insights for (candidate) disease-associated genes.

## 5. Conclusions and Discussion

Fifty years after the arrival of the Sanger sequencing technique, the sequencing technology landscape is still rapidly evolving. However, genetic diagnostic yield still varies between 40–70% for inherited HL and RD, indicating that there are still opportunities for further improvement [8,9,52]. Although novel disease-associated genes are being discovered, disease–gene identification curves are slowly reaching a plateau phase, suggesting more attention should be paid to currently missed or misinterpreted variants within known HL- or RD-associated genes that reside within complex (noncoding) regions of the genome. Recent developments of LRS techniques and optical mapping and improvements in WGS techniques offer valuable opportunities to investigate the noncoding landscape of the genome in more detail. Furthermore, the interpretation of SVs has been greatly advanced by developments in computational analysis and bioinformatics tools. Therefore, the emphasis will be on overcoming the limitations of sequencing and bioinformatic techniques in the near future. Additionally, evidence suggests that complex factors, such as modifiers, digenic inheritance, and variable penetrance, play an important role in disease-causing mechanisms in inherited HL and RD. The generation of larger, high-quality datasets will allow a better understanding of these events as well.

We foresee that, in the near future, the new technologies and improved analytical tools will reinforce the clinical diagnostic setting in order to close diagnostic gaps, as it is of utmost importance for both the affected individuals and the involved clinicians and researchers. It will help to provide guidance to affected families with regard to family planning, providing them with an optimal prognosis and counseling. In addition, with recent developments in the field of genetic therapies, the importance of genetic diagnostics can no longer be underestimated. We have come a long way from linkage analysis, starting in the early 90s, to the more recent LRS of single DNA molecules to unravel the genetic causes of HL and RD. Clinical diagnostics has significantly improved over these years, and the diagnostic yield is still increasing. We anticipate an extensive application of new technologies in the future, which will redirect traditional therapies towards precision or personalized medicine to improve treatments for HL and RD.

## Data Availability

No new data were created or analyzed in this study. Data sharing is not applicable to this article.

## References

[1] Van Camp G., Smith R. Hereditary Hearing Loss Homepage. https://hereditaryhearingloss.org/.

[2] RetNet. https://sph.uth.edu/RetNet/.

[3] World Health Organisation Deafness and Hearing Loss: Key Facts 2019 Update. https://www.who.int/news-room/fact-sheets/detail/deafness-and-hearing-loss.

[4] Mener D.J., Betz J., Genther D.J., Chen D., Lin F.R. (2013). Hearing loss and depression in older adults. J. Am. Geriatr. Soc..

[5] Sung Y.K., Li L., Blake C., Betz J., Lin F.R. (2016). Association of Hearing Loss and Loneliness in Older Adults. J. Aging Health.

[6] Shearer A.E., Hildebrand M.S., Smith R.J.H., Adam M.P., Ardinger H.H., Pagon R.A., Wallace S.E., Bean L.J.H., Mirzaa G., Amemiya A. (1993). Hereditary Hearing Loss and Deafness Overview. GeneReviews.

[7] Hartong D.T., Berson E.L., Dryja T.P. (2006). Retinitis pigmentosa. Lancet.

[8] Zazo Seco C., Wesdorp M., Feenstra I., Pfundt R., Hehir-Kwa J.Y., Lelieveld S.H., Castelein S., Gilissen C., de Wijs I.J., Admiraal R.J. (2017). The diagnostic yield of whole-exome sequencing targeting a gene panel for hearing impairment in The Netherlands. Eur. J. Hum. Genet..

[9] Haer-Wigman L., van Zelst-Stams W.A., Pfundt R., van den Born L.I., Klaver C.C., Verheij J.B., Hoyng C.B., Breuning M.H., Boon C.J., Kievit A.J. (2017). Diagnostic exome sequencing in 266 Dutch patients with visual impairment. Eur. J. Hum. Genet..

[10] Wesdorp M., Murillo-Cuesta S., Peters T., Celaya A.M., Oonk A., Schraders M., Oostrik J., Gomez-Rosas E., Beynon A.J., Hartel B.P. (2018). MPZL2, Encoding the Epithelial Junctional Protein Myelin Protein Zero-like 2, Is Essential for Hearing in Man and Mouse. Am. J. Hum. Genet..

[11] Pierrache L.H.M., Kimchi A., Ratnapriya R., Roberts L., Astuti G.D.N., Obolensky A., Beryozkin A., Tjon-Fo-Sang M.J.H., Schuil J., Klaver C.C.W. (2017). Whole-Exome Sequencing Identifies Biallelic IDH3A Variants as a Cause of Retinitis Pigmentosa Accompanied by Pseudocoloboma. Ophthalmology.

[12] McClellan J., King M.C. (2010). Genetic heterogeneity in human disease. Cell.

[13] Dawn Teare M., Barrett J.H. (2005). Genetic linkage studies. Lancet.

[14] Waters A.M., Beales P.L. (2011). Ciliopathies: An expanding disease spectrum. Pediatr. Nephrol..

[15] Cremers F.P.M., van de Pol D.J., van Kerkhoff L.P., Wieringa B., Ropers H.H. (1990). Cloning of a gene that is rearranged in patients with choroideraemia. Nature.

[16] Dryja T.P., McGee T.L., Reichel E., Hahn L.B., Cowley G.S., Yandell D.W., Sandberg M.A., Berson E.L. (1990). A point mutation of the rhodopsin gene in one form of retinitis pigmentosa. Nature.

[17] de Kok Y.J., van der Maarel S.M., Bitner-Glindzicz M., Huber I., Monaco A.P., Malcolm S., Pembrey M.E., Ropers H.H., Cremers F.P.M. (1995). Association between X-linked mixed deafness and mutations in the POU domain gene POU3F4. Science.

[18] Broadgate S., Yu J., Downes S.M., Halford S. (2017). Unravelling the genetics of inherited retinal dystrophies: Past, present and future. Prog. Retin. Eye Res..

[19] Morton N.E. (1955). Sequential tests for the detection of linkage. Am. J. Hum. Genet..

[20] Kruglyak L., Daly M.J., Reeve-Daly M.P., Lander E.S. (1996). Parametric and nonparametric linkage analysis: A unified multipoint approach. Am. J. Hum. Genet..

[21] Purcell S., Neale B., Todd-Brown K., Thomas L., Ferreira M.A., Bender D., Maller J., Sklar P., de Bakker P.I., Daly M.J. (2007). PLINK: A tool set for whole-genome association and population-based linkage analyses. Am. J. Hum. Genet..

[22] Vona B., Nanda I., Hofrichter M.A., Shehata-Dieler W., Haaf T. (2015). Non-syndromic hearing loss gene identification: A brief history and glimpse into the future. Mol. Cell Probes.

[23] Seelow D., Schuelke M., Hildebrandt F., Nürnberg P. (2009). HomozygosityMapper--an interactive approach to homozygosity mapping. Nucleic Acids Res..

[24] Woods C.G., Cox J., Springell K., Hampshire D.J., Mohamed M.D., McKibbin M., Stern R., Raymond F.L., Sandford R., Malik Sharif S. (2006). Quantification of homozygosity in consanguineous individuals with autosomal recessive disease. Am. J. Hum. Genet.

[25] Collin R.W., van den Born L.I., Klevering B.J., de Castro-Miró M., Littink K.W., Arimadyo K., Azam M., Yazar V., Zonneveld M.N., Paun C.C. (2011). High-resolution homozygosity mapping is a powerful tool to detect novel mutations causative of autosomal recessive RP in the Dutch population. Investig. Ophthalmol. Vis. Sci..

[26] Schraders M., Lee K., Oostrik J., Huygen P.L., Ali G., Hoefsloot L.H., Veltman J.A., Cremers F.P.M., Basit S., Ansar M. (2010). Homozygosity mapping reveals mutations of GRXCR1 as a cause of autosomal-recessive nonsyndromic hearing impairment. Am. J. Hum. Genet..

[27] Collin R.W.J., Littink K.W., Klevering B.J., van den Born L.I., Koenekoop R.K., Zonneveld M.N., Blokland E.A.W., Strom T.M., Hoyng C.B., den Hollander A.I. (2008). Identification of a 2 Mb human ortholog of Drosophila eyes shut/spacemaker that is mutated in patients with retinitis pigmentosa. Am. J. Hum. Genet..

[28] Messchaert M., Haer-Wigman L., Khan M.I., Cremers F.P.M., Collin R.W.J. (2018). EYS mutation update: In silico assessment of 271 reported and 26 novel variants in patients with retinitis pigmentosa. Hum. Mutat..

[29] Thiadens A.A., den Hollander A.I., Roosing S., Nabuurs S.B., Zekveld-Vroon R.C., Collin R.W., De Baere E., Koenekoop R.K., van Schooneveld M.J., Strom T.M. (2009). Homozygosity mapping reveals PDE6C mutations in patients with early-onset cone photoreceptor disorders. Am. J. Hum. Genet..

[30] Schraders M., Ruiz-Palmero L., Kalay E., Oostrik J., del Castillo F.J., Sezgin O., Beynon A.J., Strom T.M., Pennings R.J., Zazo Seco C. (2012). Mutations of the gene encoding otogelin are a cause of autosomal-recessive nonsyndromic moderate hearing impairment. Am. J. Hum. Genet..

[31] Friedman T.B., Liang Y., Weber J.L., Hinnant J.T., Barber T.D., Winata S., Arhya I.N., Asher J.H. (1995). A gene for congenital, recessive deafness DFNB3 maps to the pericentromeric region of chromosome 17. Nat. Genet..

[32] Sanger F., Nicklen S., Coulson A.R. (1977). DNA sequencing with chain-terminating inhibitors. Proc. Natl. Acad. Sci. USA.

[33] Lander E.S., Linton L.M., Birren B., Nusbaum C., Zody M.C., Baldwin J., Devon K., Dewar K., Doyle M., FitzHugh W. (2001). Initial sequencing and analysis of the human genome. Nature.

[34] Waterston R.H., Lander E.S., Sulston J.E. (2002). On the sequencing of the human genome. Proc. Natl. Acad. Sci. USA.

[35] Roberts P.J. (2001). Human genome project. Ann. Chir. Gynaecol..

[36] Venter J.C., Smith H.O., Hood L. (1996). A new strategy for genome sequencing. Nature.

[37] Schloss J.A. (2008). How to get genomes at one ten-thousandth the cost. Nat. Biotechnol..

[38] Vona B., Müller M., Dofek S., Holderried M., Löwenheim H., Tropitzsch A. (2019). A Big Data Perspective on the Genomics of Hearing Loss. Laryngorhinootologie.

[39] Levy S.E., Myers R.M. (2016). Advancements in Next-Generation Sequencing. Annu. Rev. Genom. Hum. Genet..

[40] Smith L.M., Fung S., Hunkapiller M.W., Hunkapiller T.J., Hood L.E. (1985). The synthesis of oligonucleotides containing an aliphatic amino group at the 5' terminus: Synthesis of fluorescent DNA primers for use in DNA sequence analysis. Nucleic Acids Res..

[41] Smith L.M., Sanders J.Z., Kaiser R.J., Hughes P., Dodd C., Connell C.R., Heiner C., Kent S.B., Hood L.E. (1986). Fluorescence detection in automated DNA sequence analysis. Nature.

[42] Mullis K., Faloona F., Scharf S., Saiki R., Horn G., Erlich H. (1986). Specific enzymatic amplification of DNA in vitro: The polymerase chain reaction. Cold Spring Harb. Symp. Quant. Biol..

[43] Buermans H.P., den Dunnen J.T. (2014). Next generation sequencing technology: Advances and applications. Biochim. Biophys. Acta.

[44] Choi B.Y., Park G., Gim J., Kim A.R., Kim B.J., Kim H.S., Park J.H., Park T., Oh S.H., Han K.H. (2013). Diagnostic application of targeted resequencing for familial nonsyndromic hearing loss. PLoS ONE.

[45] Dockery A., Stephenson K., Keegan D., Wynne N., Silvestri G., Humphries P., Kenna P.F., Carrigan M., Farrar G.J. (2017). Target 5000: Target Capture Sequencing for Inherited Retinal Degenerations. Genes (Basel).

[46] Khan M., Cornelis S.S., Pozo-Valero M.D., Whelan L., Runhart E.H., Mishra K., Bults F., AlSwaiti Y., AlTalbishi A., De Baere E. (2020). Resolving the dark matter of ABCA4 for 1054 Stargardt disease probands through integrated genomics and transcriptomics. Genet. Med..

[47] Lin X., Tang W., Ahmad S., Lu J., Colby C.C., Zhu J., Yu Q. (2012). Applications of targeted gene capture and next-generation sequencing technologies in studies of human deafness and other genetic disabilities. Hear. Res..

[48] Choi M., Scholl U.I., Ji W., Liu T., Tikhonova I.R., Zumbo P., Nayir A., Bakkaloğlu A., Ozen S., Sanjad S. (2009). Genetic diagnosis by whole exome capture and massively parallel DNA sequencing. Proc. Natl. Acad. Sci. USA.

[49] Stenson P.D., Ball E.V., Howells K., Phillips A.D., Mort M., Cooper D.N. (2009). The Human Gene Mutation Database: Providing a comprehensive central mutation database for molecular diagnostics and personalized genomics. Hum. Genom..

[50] Stranneheim H., Wedell A. (2016). Exome and genome sequencing: A revolution for the discovery and diagnosis of monogenic disorders. J. Intern. Med..

[51] Tucker T., Marra M., Friedman J.M. (2009). Massively parallel sequencing: The next big thing in genetic medicine. Am. J. Hum. Genet..

[52] Sloan-Heggen C.M., Bierer A.O., Shearer A.E., Kolbe D.L., Nishimura C.J., Frees K.L., Ephraim S.S., Shibata S.B., Booth K.T., Campbell C.A. (2016). Comprehensive genetic testing in the clinical evaluation of 1119 patients with hearing loss. Hum. Genet..

[53] Xu Y., Guan L., Shen T., Zhang J., Xiao X., Jiang H., Li S., Yang J., Jia X., Yin Y. (2014). Mutations of 60 known causative genes in 157 families with retinitis pigmentosa based on exome sequencing. Hum. Genet..

[54] Tiwari A., Bahr A., Bähr L., Fleischhauer J., Zinkernagel M.S., Winkler N., Barthelmes D., Berger L., Gerth-Kahlert C., Neidhardt J. (2016). Next generation sequencing based identification of disease-associated mutations in Swiss patients with retinal dystrophies. Sci. Rep..

[55] Abu-Safieh L., Alrashed M., Anazi S., Alkuraya H., Khan A.O., Al-Owain M., Al-Zahrani J., Al-Abdi L., Hashem M., Al-Tarimi S. (2013). Autozygome-guided exome sequencing in retinal dystrophy patients reveals pathogenetic mutations and novel candidate disease genes. Genome Res..

[56] Sang S., Ling J., Liu X., Mei L., Cai X., Li T., Li W., Li M., Wen J., Liu X. (2019). Proband Whole-Exome Sequencing Identified Genes Responsible for Autosomal Recessive Non-Syndromic Hearing Loss in 33 Chinese Nuclear Families. Front. Genet..

[57] Morton C.C., Nance W.E. (2006). Newborn hearing screening--a silent revolution. N. Engl. J. Med..

[58] Ellingford J.M., Barton S., Bhaskar S., Williams S.G., Sergouniotis P.I., O'Sullivan J., Lamb J.A., Perveen R., Hall G., Newman W.G. (2016). Whole Genome Sequencing Increases Molecular Diagnostic Yield Compared with Current Diagnostic Testing for Inherited Retinal Disease. Ophthalmology.

[59] Barbitoff Y.A., Polev D.E., Glotov A.S., Serebryakova E.A., Shcherbakova I.V., Kiselev A.M., Kostareva A.A., Glotov O.S., Predeus A.V. (2020). Systematic dissection of biases in whole-exome and whole-genome sequencing reveals major determinants of coding sequence coverage. Sci. Rep..

[60] Belkadi A., Bolze A., Itan Y., Cobat A., Vincent Q.B., Antipenko A., Shang L., Boisson B., Casanova J.L., Abel L. (2015). Whole-genome sequencing is more powerful than whole-exome sequencing for detecting exome variants. Proc. Natl. Acad. Sci. USA.

[61] Carss K.J., Arno G., Erwood M., Stephens J., Sanchis-Juan A., Hull S., Megy K., Grozeva D., Dewhurst E., Malka S. (2017). Comprehensive Rare Variant Analysis via Whole-Genome Sequencing to Determine the Molecular Pathology of Inherited Retinal Disease. Am. J. Hum. Genet..

[62] Wetterstrand K. DNA Sequencing Costs: Data from the NHGRI Genome Sequencing Program (GSP). www.genome.gov/sequencingcostsdata.

[63] Méjécase C., Malka S., Guan Z., Slater A., Arno G., Moosajee M. (2020). Practical guide to genetic screening for inherited eye diseases. Ther. Adv. Ophthalmol..

[64] Mantere T., Kersten S., Hoischen A. (2019). Long-Read Sequencing Emerging in Medical Genetics. Front. Genet..

[65] Eid J., Fehr A., Gray J., Luong K., Lyle J., Otto G., Peluso P., Rank D., Baybayan P., Bettman B. (2009). Real-time DNA sequencing from single polymerase molecules. Science.

[66] Magi A., Semeraro R., Mingrino A., Giusti B., D'Aurizio R. (2018). Nanopore sequencing data analysis: State of the art, applications and challenges. Brief. Bioinform..

[67] Van Dijk E.L., Jaszczyszyn Y., Naquin D., Thermes C. (2018). The Third Revolution in Sequencing Technology. Trends Genet..

[68] Schadt E.E., Turner S., Kasarskis A. (2010). A window into third-generation sequencing. Hum. Mol. Genet..

[69] Travers K.J., Chin C.S., Rank D.R., Eid J.S., Turner S.W. (2010). A flexible and efficient template format for circular consensus sequencing and SNP detection. Nucleic Acids Res..

[70] Ardui S., Ameur A., Vermeesch J.R., Hestand M.S. (2018). Single molecule real-time (SMRT) sequencing comes of age: Applications and utilities for medical diagnostics. Nucleic Acids Res..

[71] Xu M., Fujita D., Hanagata N. (2009). Perspectives and challenges of emerging single-molecule DNA sequencing technologies. Small.

[72] Chaisson M.J., Huddleston J., Dennis M.Y., Sudmant P.H., Malig M., Hormozdiari F., Antonacci F., Surti U., Sandstrom R., Boitano M. (2015). Resolving the complexity of the human genome using single-molecule sequencing. Nature.

[73] Seo J.S., Rhie A., Kim J., Lee S., Sohn M.H., Kim C.U., Hastie A., Cao H., Yun J.Y., Kim J. (2016). De novo assembly and phasing of a Korean human genome. Nature.

[74] Levene M.J., Korlach J., Turner S.W., Foquet M., Craighead H.G., Webb W.W. (2003). Zero-mode waveguides for single-molecule analysis at high concentrations. Science.

[75] Morey M., Fernández-Marmiesse A., Castiñeiras D., Fraga J.M., Couce M.L., Cocho J.A. (2013). A glimpse into past, present, and future DNA sequencing. Mol. Genet. Metab..

[76] Stoddart D., Heron A.J., Mikhailova E., Maglia G., Bayley H. (2009). Single-nucleotide discrimination in immobilized DNA oligonucleotides with a biological nanopore. Proc. Natl. Acad. Sci. USA.

[77] Kono N., Arakawa K. (2019). Nanopore sequencing: Review of potential applications in functional genomics. Dev. Growth Differ..

[78] Branton D., Deamer D.W., Marziali A., Bayley H., Benner S.A., Butler T., Di Ventra M., Garaj S., Hibbs A., Huang X. (2008). The potential and challenges of nanopore sequencing. Nat. Biotechnol..

[79] Ip C.L.C., Loose M., Tyson J.R., de Cesare M., Brown B.L., Jain M., Leggett R.M., Eccles D.A., Zalunin V., Urban J.M. (2015). MinION Analysis and Reference Consortium: Phase 1 data release and analysis. F1000Resrearch.

[80] Jain M., Koren S., Miga K.H., Quick J., Rand A.C., Sasani T.A., Tyson J.R., Beggs A.D., Dilthey A.T., Fiddes I.T. (2018). Nanopore sequencing and assembly of a human genome with ultra-long reads. Nat. Biotechnol..

[81] Mardis E.R. (2017). DNA sequencing technologies: 2006–2016. Nat. Protoc..

[82] Ebbert M.T.W., Jensen T.D., Jansen-West K., Sens J.P., Reddy J.S., Ridge P.G., Kauwe J.S.K., Belzil V., Pregent L., Carrasquillo M.M. (2019). Systematic analysis of dark and camouflaged genes reveals disease-relevant genes hiding in plain sight. Genome Biol..

[83] Reiner J., Pisani L., Qiao W., Singh R., Yang Y., Shi L., Khan W.A., Sebra R., Cohen N., Babu A. (2018). Cytogenomic identification and long-read single molecule real-time (SMRT) sequencing of a Bardet-Biedl Syndrome 9 (BBS9) deletion. NPJ Genom. Med..

[84] Bedoni N., Quinodoz M., Pinelli M., Cappuccio G., Torella A., Nigro V., Testa F., Simonelli F., Corton M., Lualdi S. (2020). An Alu-mediated duplication in NMNAT1, involved in NAD biosynthesis, causes a novel syndrome, SHILCA, affecting multiple tissues and organs. Hum. Mol. Genet..

[85] Laurent S., Gehrig C., Nouspikel T., Amr S.S., Oza A., Murphy E., Vannier A., Béna F.S., Carminho-Rodrigues M.T., Blouin J.L. (2021). Molecular characterization of pathogenic OTOA gene conversions in hearing loss patients. Hum. Mutat..

[86] Hafford-Tear N.J., Tsai Y.C., Sadan A.N., Sanchez-Pintado B., Zarouchlioti C., Maher G.J., Liskova P., Tuft S.J., Hardcastle A.J., Clark T.A. (2019). CRISPR/Cas9-targeted enrichment and long-read sequencing of the Fuchs endothelial corneal dystrophy-associated TCF4 triplet repeat. Genet. Med..

[87] Edwards H.S., Krishnakumar R., Sinha A., Bird S.W., Patel K.D., Bartsch M.S. (2019). Real-Time Selective Sequencing with RUBRIC: Read Until with Basecall and Reference-Informed Criteria. Sci. Rep..

[88] Auton A., Abecasis G.R., Altshuler D.M., Durbin R.M., Abecasis G.R., Bentley D.R., Chakravarti A., Clark A.G., Donnelly P., Eichler E.E. (2015). A global reference for human genetic variation. Nature.

[89] Richards S., Aziz N., Bale S., Bick D., Das S., Gastier-Foster J., Grody W.W., Hegde M., Lyon E., Spector E. (2015). Standards and guidelines for the interpretation of sequence variants: A joint consensus recommendation of the American College of Medical Genetics and Genomics and the Association for Molecular Pathology. Genet. Med..

[90] Eichler E.E. (2019). Genetic Variation, Comparative Genomics, and the Diagnosis of Disease. N. Engl. J. Med..

[91] Karczewski K.J., Francioli L.C., Tiao G., Cummings B.B., Alföldi J., Wang Q., Collins R.L., Laricchia K.M., Ganna A., Birnbaum D.P. (2020). The mutational constraint spectrum quantified from variation in 141,456 humans. Nature.

[92] Tucci S., Akey J.M. (2019). The long walk to African genomics. Genome Biol..

[93] Choudhury A., Aron S., Botigué L.R., Sengupta D., Botha G., Bensellak T., Wells G., Kumuthini J., Shriner D., Fakim Y.J. (2020). High-depth African genomes inform human migration and health. Nature.

[94] Boomsma D.I., Wijmenga C., Slagboom E.P., Swertz M.A., Karssen L.C., Abdellaoui A., Ye K., Guryev V., Vermaat M., van Dijk F. (2014). The Genome of the Netherlands: Design, and project goals. Eur. J. Hum. Genet..

[95] Walter K., Min J.L., Huang J., Crooks L., Memari Y., McCarthy S., Perry J.R., Xu C., Futema M., Lawson D. (2015). The UK10K project identifies rare variants in health and disease. Nature.

[96] Erikson G.A., Bodian D.L., Rueda M., Molparia B., Scott E.R., Scott-Van Zeeland A.A., Topol S.E., Wineinger N.E., Niederhuber J.E., Topol E.J. (2016). Whole-Genome Sequencing of a Healthy Aging Cohort. Cell.

[97] Rehm H.L., Berg J.S., Brooks L.D., Bustamante C.D., Evans J.P., Landrum M.J., Ledbetter D.H., Maglott D.R., Martin C.L., Nussbaum R.L. (2015). ClinGen--the Clinical Genome Resource. N. Engl. J. Med..

[98] Landrum M.J., Lee J.M., Riley G.R., Jang W., Rubinstein W.S., Church D.M., Maglott D.R. (2014). ClinVar: Public archive of relationships among sequence variation and human phenotype. Nucleic Acids Res..

[99] Fokkema I.F., Taschner P.E., Schaafsma G.C., Celli J., Laros J.F., den Dunnen J.T. (2011). LOVD v.2.0: The next generation in gene variant databases. Hum. Mutat..

[100] Stenson P.D., Mort M., Ball E.V., Evans K., Hayden M., Heywood S., Hussain M., Phillips A.D., Cooper D.N. (2017). The Human Gene Mutation Database: Towards a comprehensive repository of inherited mutation data for medical research, genetic diagnosis and next-generation sequencing studies. Hum. Genet..

[101] Azaiez H., Booth K.T., Ephraim S.S., Crone B., Black-Ziegelbein E.A., Marini R.J., Shearer A.E., Sloan-Heggen C.M., Kolbe D., Casavant T. (2018). Genomic Landscape and Mutational Signatures of Deafness-Associated Genes. Am. J. Hum. Genet..

[102] Cremers F.P.M., den Dunnen J.T., Ajmal M., Hussain A., Preising M.N., Daiger S.P., Qamar R. (2014). Comprehensive registration of DNA sequence variants associated with inherited retinal diseases in Leiden Open Variation Databases. Hum. Mutat..

[103] Cornelis S.S., Bax N.M., Zernant J., Allikmets R., Fritsche L.G., den Dunnen J.T., Ajmal M., Hoyng C.B., Cremers F.P. (2017). In Silico Functional Meta-Analysis of 5,962 ABCA4 Variants in 3,928 Retinal Dystrophy Cases. Hum. Mutat..

[104] Baux D., Blanchet C., Hamel C., Meunier I., Larrieu L., Faugère V., Vaché C., Castorina P., Puech B., Bonneau D. (2014). Enrichment of LOVD-USHbases with 152 USH2A genotypes defines an extensive mutational spectrum and highlights missense hotspots. Hum. Mutat..

[105] Astuti G.D., Bertelsen M., Preising M.N., Ajmal M., Lorenz B., Faradz S.M., Qamar R., Collin R.W., Rosenberg T., Cremers F.P. (2016). Comprehensive genotyping reveals RPE65 as the most frequently mutated gene in Leber congenital amaurosis in Denmark. Eur. J. Hum. Genet..

[106] Mackay D.S., Borman A.D., Sui R., van den Born L.I., Berson E.L., Ocaka L.A., Davidson A.E., Heckenlively J.R., Branham K., Ren H. (2013). Screening of a large cohort of leber congenital amaurosis and retinitis pigmentosa patients identifies novel LCA5 mutations and new genotype-phenotype correlations. Hum. Mutat..

[107] Ellingford J.M., Thomas H.B., Rowlands C., Arno G., Beaman G., Gomes-Silva B., Campbell C., Gossan N., Hardcastle C., Webb K. (2019). Functional and in-silico interrogation of rare genomic variants impacting RNA splicing for the diagnosis of genomic disorders. BioRxiv.

[108] Hanany M., Sharon D. (2019). Allele frequency analysis of variants reported to cause autosomal dominant inherited retinal diseases question the involvement of 19% of genes and 10% of reported pathogenic variants. J. Med. Genet..

[109] Oza A.M., DiStefano M.T., Hemphill S.E., Cushman B.J., Grant A.R., Siegert R.K., Shen J., Chapin A., Boczek N.J., Schimmenti L.A. (2018). Expert specification of the ACMG/AMP variant interpretation guidelines for genetic hearing loss. Hum. Mutat..

[110] Amberger J.S., Bocchini C.A., Schiettecatte F., Scott A.F., Hamosh A. (2015). OMIM.org: Online Mendelian Inheritance in Man (OMIM^®^), an online catalog of human genes and genetic disorders. Nucleic Acids Res..

[111] McHugh R.K., Friedman R.A. (2006). Genetics of hearing loss: Allelism and modifier genes produce a phenotypic continuum. Anat. Rec. A Discov. Mol. Cell. Evol. Biol..

[112] Cremers F.P.M., Lee W., Collin R.W.J., Allikmets R. (2020). Clinical spectrum, genetic complexity and therapeutic approaches for retinal disease caused by ABCA4 mutations. Prog. Retin. Eye Res..

[113] Runhart E.H., Khan M., Cornelis S.S., Roosing S., Del Pozo-Valero M., Lamey T.M., Liskova P., Roberts L., Stöhr H., Klaver C.C.W. (2020). Association of Sex With Frequent and Mild ABCA4 Alleles in Stargardt Disease. JAMA Ophthalmol..

[114] Runhart E.H., Valkenburg D., Cornelis S.S., Khan M., Sangermano R., Albert S., Bax N.M., Astuti G.D.N., Gilissen C., Pott J.R. (2019). Late-Onset Stargardt Disease Due to Mild, Deep-Intronic ABCA4 Alleles. Investig. Ophthalmol. Vis. Sci..

[115] Astuto L.M., Bork J.M., Weston M.D., Askew J.W., Fields R.R., Orten D.J., Ohliger S.J., Riazuddin S., Morell R.J., Khan S. (2002). CDH23 Mutation and Phenotype Heterogeneity: A Profile of 107 Diverse Families with Usher Syndrome and Nonsyndromic Deafness. Am. J. Hum. Genet..

[116] Becirovic E., Ebermann I., Nagy D., Zrenner E., Seeliger M.W., Bolz H.J. (2008). Usher syndrome type 1 due to missense mutations on both CDH23 alleles: Investigation of mRNA splicing. Hum. Mutat..

[117] Zhang L., Cheng J., Zhou Q., Khan M.A., Fu J., Duan C., Sun S., Lv H., Fu J. (2020). Targeted Next-Generation Sequencing Identified Novel Compound Heterozygous Variants in the CDH23 Gene Causing Usher Syndrome Type ID in a Chinese Patient. Front. Genet..

[118] Molina-Ramírez L.P., Lenassi E., Ellingford J.M., Sergouniotis P.I., Ramsden S.C., Bruce I.A., Black G.C.M. (2020). Establishing Genotype-phenotype Correlation in USH2A-related Disorders to Personalize Audiological Surveillance and Rehabilitation. Otol. Neurotol..

[119] Rivolta C., Sweklo E.A., Berson E.L., Dryja T.P. (2000). Missense mutation in the USH2A gene: Association with recessive retinitis pigmentosa without hearing loss. Am. J. Hum. Genet..

[120] Tatour Y., Ben-Yosef T. (2020). Syndromic Inherited Retinal Diseases: Genetic, Clinical and Diagnostic Aspects. Diagnostics (Basel).

[121] Gettelfinger J.D., Dahl J.P. (2018). Syndromic Hearing Loss: A Brief Review of Common Presentations and Genetics. J. Pediatr. Genet..

[122] Stelzer G., Rosen N., Plaschkes I., Zimmerman S., Twik M., Fishilevich S., Stein T.I., Nudel R., Lieder I., Mazor Y. (2016). The GeneCards Suite: From Gene Data Mining to Disease Genome Sequence Analyses. Curr. Protoc. Bioinform..

[123] Shen J., Scheffer D.I., Kwan K.Y., Corey D.P. (2015). SHIELD: An integrative gene expression database for inner ear research. Database (Oxford).

[124] gEAR Portal. https://umgear.org/.

[125] Ratnapriya R., Sosina O.A., Starostik M.R., Kwicklis M., Kapphahn R.J., Fritsche L.G., Walton A., Arvanitis M., Gieser L., Pietraszkiewicz A. (2019). Retinal transcriptome and eQTL analyses identify genes associated with age-related macular degeneration. Nat. Genet..

[126] Szklarczyk D., Gable A.L., Lyon D., Junge A., Wyder S., Huerta-Cepas J., Simonovic M., Doncheva N.T., Morris J.H., Bork P. (2019). STRING v11: Protein-protein association networks with increased coverage, supporting functional discovery in genome-wide experimental datasets. Nucleic Acids Res..

[127] Sobreira N., Schiettecatte F., Valle D., Hamosh A. (2015). GeneMatcher: A matching tool for connecting investigators with an interest in the same gene. Hum. Mutat..

[128] The European Retinal Disease Consortium (ERDC). www.ERDC.info.

[129] Di Stazio M., Morgan A., Brumat M., Bassani S., Dell'Orco D., Marino V., Garagnani P., Giuliani C., Gasparini P., Girotto G. (2020). New age-related hearing loss candidate genes in humans: An ongoing challenge. Gene.

[130] Astuti G.D.N., van den Born L.I., Khan M.I., Hamel C.P., Bocquet B., Manes G., Quinodoz M., Ali M., Toomes C., McKibbin M. (2018). Identification of Inherited Retinal Disease-Associated Genetic Variants in 11 Candidate Genes. Genes (Basel).

[131] Collins R.L., Brand H., Karczewski K.J., Zhao X., Alföldi J., Francioli L.C., Khera A.V., Lowther C., Gauthier L.D., Wang H. (2020). A structural variation reference for medical and population genetics. Nature.

[132] Littink K.W., Pott J.W., Collin R.W., Kroes H.Y., Verheij J.B., Blokland E.A., de Castro Miró M., Hoyng C.B., Klaver C.C., Koenekoop R.K. (2010). A novel nonsense mutation in CEP290 induces exon skipping and leads to a relatively mild retinal phenotype. Investig. Ophthalmol. Vis. Sci..

[133] Roosing S., Cremers F.P.M., Riemslag F.C.C., Zonneveld-Vrieling M.N., Talsma H.E., Klessens-Godfroy F.J.M., den Hollander A.I., van den Born L.I. (2017). A Rare Form of Retinal Dystrophy Caused by Hypomorphic Nonsense Mutations in CEP290. Genes (Basel).

[134] DiStefano M.T., Hemphill S.E., Cushman B.J., Bowser M.J., Hynes E., Grant A.R., Siegert R.K., Oza A.M., Gonzalez M.A., Amr S.S. (2018). Curating Clinically Relevant Transcripts for the Interpretation of Sequence Variants. J. Mol. Diagn..

[135] Kircher M., Witten D.M., Jain P., O'Roak B.J., Cooper G.M., Shendure J. (2014). A general framework for estimating the relative pathogenicity of human genetic variants. Nat. Genet..

[136] Grantham R. (1974). Amino Acid Difference Formula to Help Explain Protein Evolution. Science.

[137] Schwarz J.M., Cooper D.N., Schuelke M., Seelow D. (2014). MutationTaster2: Mutation prediction for the deep-sequencing age. Nat. Methods.

[138] Pollard K.S., Hubisz M.J., Rosenbloom K.R., Siepel A. (2010). Detection of nonneutral substitution rates on mammalian phylogenies. Genome Res..

[139] Adzhubei I.A., Schmidt S., Peshkin L., Ramensky V.E., Gerasimova A., Bork P., Kondrashov A.S., Sunyaev S.R. (2010). A method and server for predicting damaging missense mutations. Nat. Methods.

[140] Vaser R., Adusumalli S., Leng S.N., Sikic M., Ng P.C. (2015). SIFT missense predictions for genomes. Nature Protocols.

[141] Desmet F.-O., Hamroun D., Lalande M., Collod-Béroud G., Claustres M., Béroud C. (2009). Human Splicing Finder: An online bioinformatics tool to predict splicing signals. Nucleic Acids Res..

[142] Shapiro M.B., Senapathy P. (1987). RNA splice junctions of different classes of eukaryotes: Sequence statistics and functional implications in gene expression. Nucleic Acids Res..

[143] Yeo G., Burge C.B. (2004). Maximum entropy modeling of short sequence motifs with applications to RNA splicing signals. J. Comput. Biol..

[144] Pertea M., Lin X., Salzberg S.L. (2001). GeneSplicer: A new computational method for splice site prediction. Nucleic Acids Res..

[145] Reese M.G., Eeckman F.H., Kulp D., Haussler D. (1997). Improved splice site detection in Genie. J. Comput. Biol..

[146] Jaganathan K., Kyriazopoulou Panagiotopoulou S., McRae J.F., Darbandi S.F., Knowles D., Li Y.I., Kosmicki J.A., Arbelaez J., Cui W., Schwartz G.B. (2019). Predicting Splicing from Primary Sequence with Deep Learning. Cell.

[147] Sangermano R., Khan M., Cornelis S.S., Richelle V., Albert S., Garanto A., Elmelik D., Qamar R., Lugtenberg D., van den Born L.I. (2018). ABCA4 midigenes reveal the full splice spectrum of all reported noncanonical splice site variants in Stargardt disease. Genome Res..

[148] Collin R.W., de Heer A.M., Oostrik J., Pauw R.J., Plantinga R.F., Huygen P.L., Admiraal R., de Brouwer A.P., Strom T.M., Cremers C.W. (2008). Mid-frequency DFNA8/12 hearing loss caused by a synonymous TECTA mutation that affects an exonic splice enhancer. Eur. J. Hum. Genet..

[149] Riepe T., Khan M., Roosing S., Cremers F.P.M., ‘t Hoen P. (2020). Benchmarking deep learning splice prediction tools using functional splice assays. Authorea Prepr..

[150] Rowlands C.F., Baralle D., Ellingford J.M. (2019). Machine Learning Approaches for the Prioritization of Genomic Variants Impacting Pre-mRNA Splicing. Cells.

[151] Cherry T.J., Yang M.G., Harmin D.A., Tao P., Timms A.E., Bauwens M., Allikmets R., Jones E.M., Chen R., De Baere E. (2020). Mapping the cis-regulatory architecture of the human retina reveals noncoding genetic variation in disease. Proc. Natl. Acad. Sci. USA.

[152] Van der Lee R., Correard S., Wasserman W.W. (2020). Deregulated Regulators: Disease-Causing cis Variants in Transcription Factor Genes. Trends Genet..

[153] Lupiáñez D.G., Kraft K., Heinrich V., Krawitz P., Brancati F., Klopocki E., Horn D., Kayserili H., Opitz J.M., Laxova R. (2015). Disruptions of topological chromatin domains cause pathogenic rewiring of gene-enhancer interactions. Cell.

[154] De Kok Y.J.M., Vossenaar E.R., Cremers C.W.R.J., Dahl N., Laporte J., Jia Hu L., Lacombe D., Fischel-Ghodsian N., Friedman R.A., Parnes L.S. (1996). Identification of a Hot Spot for Microdeletions in Patients with X-linked Deafness Type 3 (DFN3) 900 kb Proximal to the DFN3 gene POU3F4. Hum. Mol. Genet..

[155] Naranjo S., Voesenek K., de la Calle-Mustienes E., Robert-Moreno A., Kokotas H., Grigoriadou M., Economides J., Van Camp G., Hilgert N., Moreno F. (2010). Multiple enhancers located in a 1-Mb region upstream of POU3F4 promote expression during inner ear development and may be required for hearing. Hum. Genet..

[156] Fornes O., Castro-Mondragon J.A., Khan A., van der Lee R., Zhang X., Richmond P.A., Modi B.P., Correard S., Gheorghe M., Baranašić D. (2020). JASPAR 2020: Update of the open-access database of transcription factor binding profiles. Nucleic Acids Res..

[157] Perez-Cervantes C., Smith L.A., Nadadur R.D., Hughes A.E.O., Wang S., Corbo J.C., Cepko C., Lonfat N., Moskowitz I.P. (2020). Enhancer transcription identifies cis-regulatory elements for photoreceptor cell types. Development.

[158] Davis C.A., Hitz B.C., Sloan C.A., Chan E.T., Davidson J.M., Gabdank I., Hilton J.A., Jain K., Baymuradov U.K., Narayanan A.K. (2018). The Encyclopedia of DNA elements (ENCODE): Data portal update. Nucleic Acids Res..

[159] Fishilevich S., Nudel R., Rappaport N., Hadar R., Plaschkes I., Iny Stein T., Rosen N., Kohn A., Twik M., Safran M. (2017). GeneHancer: Genome-wide integration of enhancers and target genes in GeneCards. Database (Oxford).

[160] Gao T., Qian J. (2020). EnhancerAtlas 2.0: An updated resource with enhancer annotation in 586 tissue/cell types across nine species. Nucleic Acids Res..

[161] de Bruijn S.E., Fiorentino A., Ottaviani D., Fanucchi S., Melo U.S., Corral-Serrano J.C., Mulders T., Georgiou M., Rivolta C., Pontikos N. (2020). Structural Variants Create New Topological-Associated Domains and Ectopic Retinal Enhancer-Gene Contact in Dominant Retinitis Pigmentosa. Am. J. Hum. Genet..

[162] Lizio M., Abugessaisa I., Noguchi S., Kondo A., Hasegawa A., Hon C.C., de Hoon M., Severin J., Oki S., Hayashizaki Y. (2018). Update of the FANTOM web resource: Expansion to provide additional transcriptome atlases. Nucleic Acids Res..

[163] Brandt T., Sack L.M., Arjona D., Tan D., Mei H., Cui H., Gao H., Bean L.J.H., Ankala A., Del Gaudio D. (2020). Adapting ACMG/AMP sequence variant classification guidelines for single-gene copy number variants. Genet Med..

[164] Dixon J.R., Selvaraj S., Yue F., Kim A., Li Y., Shen Y., Hu M., Liu J.S., Ren B. (2012). Topological domains in mammalian genomes identified by analysis of chromatin interactions. Nature.

[165] Spielmann M., Lupiáñez D.G., Mundlos S. (2018). Structural variation in the 3D genome. Nat. Rev. Genet..

[166] Franke M., Ibrahim D.M., Andrey G., Schwarzer W., Heinrich V., Schöpflin R., Kraft K., Kempfer R., Jerković I., Chan W.L. (2016). Formation of new chromatin domains determines pathogenicity of genomic duplications. Nature.

[167] Ibrahim D.M., Mundlos S. (2020). Three-dimensional chromatin in disease: What holds us together and what drives us apart?. Curr. Opin. Cell Biol..

[168] Van Schil K., Naessens S., Van de Sompele S., Carron M., Aslanidis A., Van Cauwenbergh C., Kathrin Mayer A., Van Heetvelde M., Bauwens M., Verdin H. (2018). Mapping the genomic landscape of inherited retinal disease genes prioritizes genes prone to coding and noncoding copy-number variations. Genet. Med..

[169] Shearer A.E., Kolbe D.L., Azaiez H., Sloan C.M., Frees K.L., Weaver A.E., Clark E.T., Nishimura C.J., Black-Ziegelbein E.A., Smith R.J. (2014). Copy number variants are a common cause of non-syndromic hearing loss. Genome Med..

[170] Nikopoulos K., Cisarova K., Quinodoz M., Koskiniemi-Kuendig H., Miyake N., Farinelli P., Rehman A.U., Khan M.I., Prunotto A., Akiyama M. (2019). A frequent variant in the Japanese population determines quasi-Mendelian inheritance of rare retinal ciliopathy. Nat. Commun..

[171] Yan D., Liu X.-Z. (2010). Modifiers of hearing impairment in humans and mice. Curr. Genom..

[172] Norman C.S., O’Gorman L., Gibson J., Pengelly R.J., Baralle D., Ratnayaka J.A., Griffiths H., Rose-Zerilli M., Ranger M., Bunyan D. (2017). Identification of a functionally significant tri-allelic genotype in the Tyrosinase gene (TYR) causing hypomorphic oculocutaneous albinism (OCA1B). Sci. Rep..

[173] Grønskov K., Jespersgaard C., Bruun G.H., Harris P., Brøndum-Nielsen K., Andresen B.S., Rosenberg T. (2019). A pathogenic haplotype, common in Europeans, causes autosomal recessive albinism and uncovers missing heritability in OCA1. Sci. Rep..

[174] Green D.J., Sallah S.R., Ellingford J.M., Lovell S.C., Sergouniotis P.I. (2020). Variability in Gene Expression is Associated with Incomplete Penetrance in Inherited Eye Disorders. Genes (Basel).

[175] Llavona P., Pinelli M., Mutarelli M., Marwah V.S., Schimpf-Linzenbold S., Thaler S., Yoeruek E., Vetter J., Kohl S., Wissinger B. (2017). Allelic Expression Imbalance in the Human Retinal Transcriptome and Potential Impact on Inherited Retinal Diseases. Genes (Basel).

[176] Runhart E.H., Sangermano R., Cornelis S.S., Verheij J., Plomp A.S., Boon C.J.F., Lugtenberg D., Roosing S., Bax N.M., Blokland E.A.W. (2018). The Common ABCA4 Variant p.Asn1868Ile Shows Nonpenetrance and Variable Expression of Stargardt Disease When Present in trans With Severe Variants. Investig. Ophthalmol. Vis. Sci..

[177] Smits J.J., van Beelen E., Weegerink N.J.D., Oostrik J., Huygen P.L.M., Beynon A.J., Lanting C.P., Kunst H.P.M., Schraders M., Kremer H. (2020). A Novel COCH Mutation Affects the vWFA2 Domain and Leads to a Relatively Mild DFNA9 Phenotype. Otol. Neurotol..

[178] Vithana E.N., Abu-Safieh L., Pelosini L., Winchester E., Hornan D., Bird A.C., Hunt D.M., Bustin S.A., Bhattacharya S.S. (2003). Expression of PRPF31 mRNA in patients with autosomal dominant retinitis pigmentosa: A molecular clue for incomplete penetrance?. Investig. Ophthalmol. Vis. Sci..

[179] de Bruijn S.E., Smits J.J., Liu C., Lanting C.P., Beynon A.J., Blankevoort J., Oostrik J., Koole W., de Vrieze E., Cremers C.W.R.J. (2021). A RIPOR2 in-frame deletion is a frequent and highly penetrant cause of adult-onset hearing loss. J. Med Genet..

[180] Yauy K., de Leeuw N., Yntema H.G., Pfundt R., Gilissen C. (2020). Accurate detection of clinically relevant uniparental disomy from exome sequencing data. Genet. Med..

[181] Fingert J.H., Eliason D.A., Phillips N.C., Lotery A.J., Sheffield V.C., Stone E.M. (2006). Case of Stargardt disease caused by uniparental isodisomy. Arch. Ophthalmol..

[182] Alvarez A., del Castillo I., Pera A., Villamar M., Moreno-Pelayo M.A., Rivera T., Solanellas J., Moreno F. (2003). Uniparental disomy of chromosome 13q causing homozygosity for the 35delG mutation in the gene encoding connexin26 (GJB2) results in prelingual hearing impairment in two unrelated Spanish patients. J. Med. Genet..

[183] Fu J., Shen S., Cheng J., Lv H., Fu J. (2020). A case of Usher syndrome type IIA caused by a rare USH2A homozygous frameshift variant with maternal uniparental disomy (UPD) in a Chinese family. J. Cell. Mol. Med..

[184] Morgan A., Lenarduzzi S., Cappellani S., Pecile V., Morgutti M., Orzan E., Ghiselli S., Ambrosetti U., Brumat M., Gajendrarao P. (2018). Genomic Studies in a Large Cohort of Hearing Impaired Italian Patients Revealed Several New Alleles, a Rare Case of Uniparental Disomy (UPD) and the Importance to Search for Copy Number Variations. Front. Genet..

[185] Slijkerman R.W., Song F., Astuti G.D., Huynen M.A., van Wijk E., Stieger K., Collin R.W. (2015). The pros and cons of vertebrate animal models for functional and therapeutic research on inherited retinal dystrophies. Prog. Retin. Eye Res..

[186] Dickinson M.E., Flenniken A.M., Ji X., Teboul L., Wong M.D., White J.K., Meehan T.F., Weninger W.J., Westerberg H., Adissu H. (2016). High-throughput discovery of novel developmental phenotypes. Nature.

[187] Vona B., Doll J., Hofrichter M.A.H., Haaf T., Varshney G.K. (2020). Small fish, big prospects: Using zebrafish to unravel the mechanisms of hereditary hearing loss. Hear. Res..

[188] Tang P.C., Hashino E., Nelson R.F. (2020). Progress in Modeling and Targeting Inner Ear Disorders with Pluripotent Stem Cells. Stem Cell Rep..

[189] Kruczek K., Swaroop A. (2020). Pluripotent stem cell-derived retinal organoids for disease modeling and development of therapies. Stem Cells.

[190] Vissers L.E., Veltman J.A., van Kessel A.G., Brunner H.G. (2005). Identification of disease genes by whole genome CGH arrays. Hum. Mol. Genet..

[191] Cui C., Shu W., Li P. (2016). Fluorescence In situ Hybridization: Cell-Based Genetic Diagnostic and Research Applications. Front. Cell Dev. Biol..

[192] Hyon C. (2017). Usefulness of CGH-array and SNP-array for the etiological diagnosis of premature ovarian insufficiency. Biol. Aujourdhui.

[193] Mantere T., Neveling K., Pebrel-Richard C., Benoist M., van der Zande G., Kater-Baats E., Baatout I., van Beek R., Yammine T., Oorsprong M. (2020). Next generation cytogenetics: Genome-imaging enables comprehensive structural variant detection for 100 constitutional chromosomal aberrations in 85 samples. BioRxiv.

[194] Lee C.N., Lin S.Y., Lin C.H., Shih J.C., Lin T.H., Su Y.N. (2012). Clinical utility of array comparative genomic hybridisation for prenatal diagnosis: A cohort study of 3171 pregnancies. BJOG.

[195] Yuan Y., Chung C.Y., Chan T.F. (2020). Advances in optical mapping for genomic research. Comput. Struct. Biotechnol. J..

[196] Chan S., Lam E., Saghbini M., Bocklandt S., Hastie A., Cao H., Holmlin E., Borodkin M. (2018). Structural Variation Detection and Analysis Using Bionano Optical Mapping. Methods Mol. Biol..

[197] Chen M., Zhang M., Qian Y., Yang Y., Sun Y., Liu B., Wang L., Dong M. (2020). Identification of a likely pathogenic structural variation in the LAMA1 gene by Bionano optical mapping. NPJ Genom. Med..

[198] Cummings B.B., Marshall J.L., Tukiainen T., Lek M., Donkervoort S., Foley A.R., Bolduc V., Waddell L.B., Sandaradura S.A., O'Grady G.L. (2017). Improving genetic diagnosis in Mendelian disease with transcriptome sequencing. Sci. Transl. Med..

[199] Kremer L.S., Bader D.M., Mertes C., Kopajtich R., Pichler G., Iuso A., Haack T.B., Graf E., Schwarzmayr T., Terrile C. (2017). Genetic diagnosis of Mendelian disorders via RNA sequencing. Nat. Commun..

[200] Rosenbloom K.R., Sloan C.A., Malladi V.S., Dreszer T.R., Learned K., Kirkup V.M., Wong M.C., Maddren M., Fang R., Heitner S.G. (2013). ENCODE data in the UCSC Genome Browser: Year 5 update. Nucleic Acids Res..

[201] Ray T.A., Cochran K., Kozlowski C., Wang J., Alexander G., Cady M.A., Spencer W.J., Ruzycki P.A., Clark B.S., Laeremans A. (2020). Comprehensive identification of mRNA isoforms reveals the diversity of neural cell-surface molecules with roles in retinal development and disease. Nat. Commun..

[202] Single Cell Portal (Broad Institute). https://singlecell.broadinstitute.org/single_cell.

